# Anti-HIV Activity of Tigliane Derivatives from *Euphorbia nicaeensis* Roots

**DOI:** 10.3390/molecules30071452

**Published:** 2025-03-25

**Authors:** Gordana Krstić, Milka Jadranin, Dominique Schols, Sandra Claes, Vele Tešević, Boris Mandić, Slobodan Milosavljević, Karlo Wittine

**Affiliations:** 1 University of Belgrade—Faculty of Chemistry, Studentski trg 12–16, 11010 Belgrade, Serbia; vtesevic@chem.bg.ac.rs (V.T.); borism@chem.bg.ac.rs (B.M.); smilo@chem.bg.ac.rs (S.M.); 2 Department of Chemistry, University of Belgrade—Institute of Chemistry, Technology and Metallurgy, Njegoševa 12, 11000 Belgrade, Serbia; milka.jadranin@ihtm.bg.ac.rs; 3 Translational Virology, Laboratory of Molecular, Structural and Rega Institute, Herestraat 49, 3000 Leuven, Belgium; dominique.schols@kuleuven.be (D.S.); sandra.claes@kuleuven.be (S.C.); 4 Serbian Academy of Science and Arts, Kneza Mihaila 35, 11000 Belgrade, Serbia; 5 Selvita Ltd., Prilaz baruna Filipovića 29, 10000 Zagreb, Croatia; 6 Faculty of Biotechnology and Drug Development, University of Rijeka, Radmile Matejčić 2, 51000 Rijeka, Croatia

**Keywords:** *Euphorbia*, *Euphorbia nicaeensis*, tigliane, anti-HIV

## Abstract

Five previously undescribed tigliane diterpenes (**1**–**4** and **7**), along with three known tiglianes (**5**, **6,** and **8**) were isolated from the root extract of *Euphorbia nicaeensis* using chromatographic techniques. The structures of the isolated compounds were determined using spectroscopic techniques. The isolated compounds were tested for anti-HIV activity against HIV-1 NL4.3 and HIV-2 ROD strains. Two derivatives (**2** and **8**) exhibited significant anti-HIV activity, with IC_50_ values ranging from 1.10 to 7.47 µM. This study highlights the potential of *E. nicaeensis* root as a source of novel bioactive tigliane derivatives, warranting further investigation for possible use in HIV treatment.

## 1. Introduction

With more than 2000 registered species growing worldwide, *Euphorbia* plants represent a vast reservoir of biologically active molecules [1,2,3]. Over the years, numerous terpenes, including sesquiterpenes, diterpenes, triterpenes, and various phenolic compounds, have been isolated from these species. The use of *Euphorbia* species in traditional medicine is widespread, particularly in the East, though it is also common to other parts of the world. Various plant parts, such as latex, leaves, roots, and fruits are utilized, as many of the compounds isolated thus far have demonstrated significant biological activities, including antibacterial, antiviral, and antitumor effects [4]. Tigliane diterpenes, the subject of this study, are among the most active principles of the family and have received considerable attention so far [4,5]. However, some of these compounds can be highly toxic and act as tumor promoters [6]. Thus, one prominent class of tigliane diterpenes, phorbols, is known to exhibit both, a tumor-promoting activity and strong anti-HIV properties [3,6,7]. The type of activity expressed depends on several factors, particularly the way rings A and B are bound in the tigliane structure and the position and type of ester groups attached to the phorbol skeleton [7]. One of the best known tigliane exhibiting considerable anti-HIV activity is non-tumor promoting phorbol ester prostratin [8], isolated from the poisonous plant *Pimelea prostrata* (Thymelaeaceae), native to New Zealand [9]. After numerous studies, prostratin has been advanced into phase I human clinical trials for the treatment of HIV/AIDS [10]. This prompted the numerous studies of antiviral potential of a number of tiglianes from other natural sources [11,12,13] as well as the synthetic ones [5] with the aim to enhance knowledge of the structure–activity relationship (SARS) of these classes.

Previous phytochemical studies of *E. nicaeensis* involved epicuticular wax constituents [14], tetracyclic triterpenoids [15,16], glucocerebrosides [17] activity and glyceroglycolipids with anti-inflammatory activity [18], jatrophane diterpenoids from latex [19], and roots [20] exhibiting anticancer activities. As a continuation of our search for bioactive compounds from the species belonging to the genus *Euphorbia,* we now report the isolation of eight tigliane diterpenes (**1**–**8**) from the roots of *E. nicaeensis* and evaluation of their anti-HIV activity.

## 2. Results and Discussion

**Figure 1:**
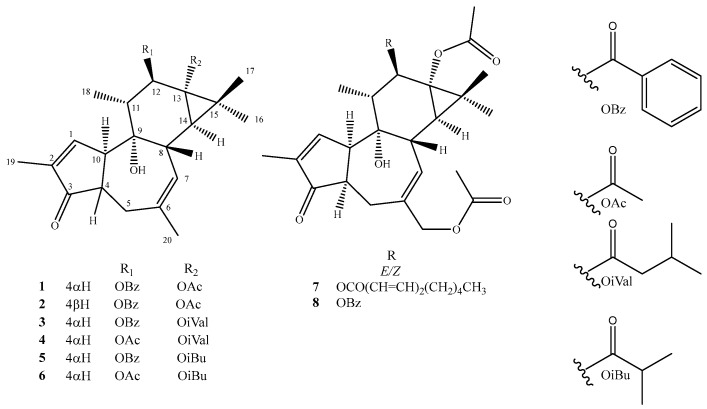
Structures of the isolated compounds (**1**–**8**).

Eight compounds were isolated by purification of the ethanolic extract of *E. nicaeensis* roots using a combination of different chromatographic techniques. The structure determination of these compounds was carried out by spectroscopic analysis (1D and 2D NMR), as well as HRESIMS experiments, and by comparison of the spectral data to those previously published for the related compounds. In this way, it was established that all isolated compounds are tigliane diterpenes, of which three tiglianes were previously known (12β-benzoyloxy-13α-isobutanoyloxy-4-epi-4,20-dideoxyphforbol, **5** [21], 12β-acetyloxy-13α-isobutanoyloxy-4-epi-4,20-dideoxyphorbol, **6** [22] and 13α,20-diacetylohy-12β-benzoyloxy-4-epi-4-deoxyphorbol, **8** [23]), while five tiglianes were not previously described (**1**–**4** and **7**) (Figure 1). The NMR spectral data of the previously undescribed compounds are listed in Table 1 and Table 2.

**Figure 2:**
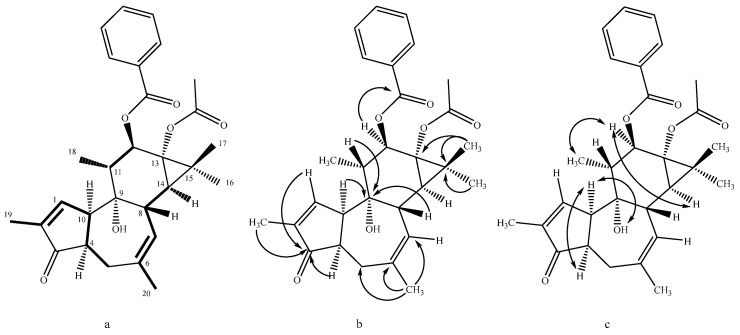
Key correlations in (**a**) COSY (three spin systems, bold), (**b**) HMBC and (**c**) NOESY spectrum of **1**.

Nicaeenin H (**1**) was isolated as a colorless amorphous substance. Its HRESIMS spectrum exhibited [M + Na]^+^ ion at *m*/*z* 501.2246 corresponding to the molecular formula C_29_H_34_O_6_ (calcd. for C_29_H_34_NaO_6_, 501.2248). Most of the ^1^H and ^13^C NMR data (Table 1 and Table 2) of **1** were almost identical to those of co-occurring known phorbol derivative **5** [21]. The only difference between the NMR spectra of **1** and **5** was that concerning 13-ester substituent. Instead of the signals typical for the isobutyrate group observed in **5**, the NMR spectra of **1** contained resonances typical for the acetate moiety (δ_C_ 173.5 and 21.1, δ_H_ 2.09 s, 3H). This, together with the evidence provided by COSY, HMBC, and NOESY spectra (Figure 2) agreed with 4,20-dideoxy-4α-phorbol-12-benzoate-13-acetate structure of **1**.

Nicaeenin I (**2**) was isolated as a colorless amorphous substance with the molecular formula C_29_H_34_O_6_, according to the HRESIMS peak at *m*/*z* 479.2416 [M + H]^+^ (calcd. for C_29_H_35_O_6_, 479.2428), the same as that of **1**. A detailed analysis of the 1D and 2D NMR spectra indicated the same skeleton as in **1**, as well as the same 12-benzoyloxy and 13-acetoxy moieties. The main differences were those concerning NMR data (Table 1 and Table 2) of the A and B rings, thus suggesting a different stereochemistry of the A/B ring junction. The NMR data of C(4)H-C(10)H moiety in **2** which are diagnostic for the stereochemistry of the A/B ring junction [15], i.e., δ_H_ 2.49 (H-4), 3.32 (H-10), δ_C_ 44.8 (C-4) and 54.6 (C-10), were in agreement with 4βH,10αH (*trans*) configuration for this compound. The close similarity of the ^1^H and ^13^C NMR chemical shifts of these nuclei with those of the related 4,20-dideoxyphorbols [10] was in accordance with the proposed *trans* A/B (i.e., 4*β*H,10αH) stereochemistry in **2**. The NOE correlations H-4β/H-5β (δ_H_ 2.86), H-4β/H-8β (δ_H_ 2.86), and H-4β/H-11β (δ_H_ 1.72) also supported this stereochemical assignment, thus indicating a structure of 4,20-dideoxy-phorbol-12-benzoate-13-acetate.

Nicaeenin J (**3**) was isolated as a colorless amorphous substance with the molecular formula C_32_H_40_O_6_, as established by [M + Na]^+^ ion at *m*/*z* 543.2714 HRESIMS (calcd. for C_32_H_40_NaO_6_, 543.2717). The spectral data of **3** indicated the same skeleton and stereochemistry as in **1** (Table 1 and Table 2), with the only difference concerning the ester attached at the C-13 position. The molecular mass of **3**, higher than that of **1** (Δ M = 42 Daltons), together with the occurrence of the ^1^H NMR signals, typical for isovalerate ester, i.e., δ_H_ 2.21, 2H m, δ_H_ 2.10, 1H m, δ_H_ 0.97, 3H d, and 0.95, 3H d, indicated the structure of 4,20-dideoxy-4α-phorbol-12-benzoate-13-isovalerate for **3**.

Nicaeenin K (**4**), a colorless amorphous substance, with the molecular formula C_27_H_40_O_6_, based on the HRESIMS peak at *m*/*z* 481,2542 [M + Na]^+^ (calcd. for C_27_H_38_NaO_6_, 481,2561) showed a great similarity with the NMR spectra of **3** (Table 1 and Table 2). Instead of the 12-benzoate ester detected in **3**, compound **4** contained an acetate group (δ_H_ 2.11, 3H, s; δ_C_ 170.8, 21.2). The HMBC correlation of the acetate carbonyl with H-12α (δ_H_ 5.43, d, *J* = 10 Hz), as well as NOE between the isovalerate methyls (δ_H_ 0.96 and 0.94) and H-12α, indicated a 12-acetate-13-izovalerate structure. The chemical shifts of H-4 (δ_H_ 2.68), H-10 (δ_H_ 3.32), C-4 (δ_C_ 49.4), and (δ_C_ 47.2) were in agreement with the 4αH,10αH (*cis*) configuration, thus indicating the structure of 4,20-dideoxy-4α-phorbol-12-acetate-13-isovalerate.

Nicaeenin L (**7**), a colorless amorphous substance, exhibited molecular formula C_34_H_46_O_8_, according to HRESIMS ion at *m*/*z* 583.3265 [M + H]^+^ (calcd. for C_34_H_47_O_8_, 583.3265). The ^1^H and ^13^C NMR spectra of **7** (Table 1 and Table 2, assigned using 2D NMR methods) displayed a very similar pattern as the co-occurring known 4-deoxy-4*α*-phorbol-12-benzoate-20,13-diacetate (**8**) [23]. The main difference between **7** and **8** was that concerning 12-ester residue. Instead of the low-field aromatic NMR signals of the benzoate functionality observed in **8**, the ^1^H and ^13^C NMR spectra of **7** contained signals typical for the 2*E*,4*Z*-decadienoate group (Table 1 and Table 2), assigned by close similarity of NMR spectral data with that of diterpenes from *Euphorbia kansui* containing the same ester group [24], as well as the MS data. The chemical shifts of H-4 (δ_H_ 2.75), H-10 (δ_H_ 3.47), C-4 (δ_C_ 49.1), and (δ_C_ 47.1) were in accordance with 4αH,10αH (*cis*) configuration in **7**. The HMBC correlations H-12 (δ_H_ 5.55)/C-1′ (δ_C_ 167.2), H_2_-20 (δ_H_ 4.41)/C-1“’ (δ_C_ 173.7) and the chemical shift of C-13 (δ_C_ 65.5) fully supported the proposed 4-deoxy-4α-phorbol-12-(2*E*,4*Z*-decadienoate)-20,13-diacetate structure.

After isolation, purification, and structure determination, anti-HIV activity of isolated compounds was evaluated. Compounds **1**, **3**, **4**, **5**, and **6** showed IC₅₀ values greater than their respective CC₅₀ values, meaning they are not effective at inhibiting HIV-1 replication within a safe concentration range. Also, these compounds display poor activity against HIV-2 (ROD). The most promising derivatives, **2** and **8**, inhibited both HIV-1 and HIV-2 replication in MT-4 cells. The IC_50_ values of **2** were 7.5 µM and 1.7 µM for HIV-1 and HIV-2, respectively, while these values for **8** were 3.3 and 1.1 µM. The IC_50_ values of the active metabolites were around 7 to 55 times lower than the 50% cytotoxic concentration (CC_50_) (Table 3). The well-described compounds PMPA (tenofovir) and AMD3100 (plerixafor) were used as reference compounds in the anti-HIV replication assay (Table 3). ANOVA (Excel) was used for statistical analysis, and no significant differences were found between the IC_50_ of HIV-1 and HIV-2, and CC_50_ data (*p*-value = 0.1591). Since the *p*-value is greater than 0.05, it indicates that there is no statistically significant difference among these three data groups.

Based on the results presented in Table 3, a preliminary structure–activity relationship for some isolated compounds was determined. Specifically, a comparison of compounds **1** and **2** showed that the 4β could significantly enhance the anti-HIV activity, and comparison of compounds **1** and **3** suggested that the presence of the iVal group reduces the anti-HIV activity, while comparing compounds **1** and **5**, as well as compounds **3** and **5,** revealed that the iBu group increases the anti-HIV activity. Furthermore, a pairwise comparison of compounds **3** and **4**, as well as compounds **5** and **6**, indicated that replacing the OBz group with OAc results in a decrease in anti-HIV activity, highlighting the importance of the OBz group for maintaining activity. On the other hand, when we compare the structures and anti-HIV-1 activities of the isolated compounds with the activities of other tiglianes, such as stelleracin C, isolated from *Reutealis trisperma*, *Stellera chamaejasme*, *Wistroemia scytophylla*, and *Wikstroemia lamatsoensis* [25,26,27,28], and 12-deoxyphorbol-13-hexadecanoate, isolated from *Reutealis trisperma* and *Euphorbia fischeriana* [25,29], whose IC_50_ values are 0.0023 and 0.022 µM, respectively, we can conclude that the presence of fatty acid esters at the C-12 position significantly enhances anti-HIV-1 activity and lowers IC_50_ values. The presence of fatty acid esters facilitates easier molecular entry into the cell and increases the concentration of the molecule at the site of action.

These findings suggest that the root of *E. nicaeensis* may be a promising source of rare metabolites with notable anti-HIV activity. Further preclinical studies are needed to fully assess the safety and efficacy of these metabolites.

## 3. Materials and Methods

### 3.1. General Experimental Procedures

Optical rotations were measured on an Autopol IV (Rudolph Research Analytical, Hackettstown, NJ, USA) polarimeter equipped with a sodium lamp (589 nm) and 10 cm microcell. All NMR data were acquired on Bruker Avance III 500 NMR spectrometer (500 MHz for ^1^H and 125 MHz for ^13^C NMR, in CDCl_3_, with TMS as internal standard) (Bruker, Billerica, MA, USA). The NMR spectra were analyzed using TopSpin 3.6.2 software. High-resolution LC/ESI positive TOF mass spectra were measured on a HPLC instrument (Agilent 1200 Series) coupled with a 6210 Time-of-Flight LC/MS system (Agilent Technologies, Santa Clara, CA, USA). NP-HPLC-DAD: Agilent Technologies 1260 Series liquid chromatograph equipped with diode-array detector, autosampler, and collector; Zorbax RX-Sil (250 × 9.4 mm; 5 μm) column (Agilent Technologies, Waldbronn, Germany). RP-HPLC-DAD: Agilent Technologies 1100 Series liquid chromatograph (Agilent Technologies, Waldbronn, Germany) equipped with diode-array detector, autosampler, and collector; Zorbax XDB-C18 column (250 × 9.4 mm; 5 μm) (Agilent Technologies, Waldbronn, Germany). Dry-column flash chromatography (DCFC) was performed on silica gel (ICN Silica 12–26 60 Å, Merck, Darmstadt, Germany) [21]. Silica gel 60 F_254_ precoated aluminium sheets (0.25 mm, Merck) for TLC control were used. The TLC plates were visualized under a UV lamp at 254 nm and detected by spraying with solution of cerium molybdate in sulphuric acid, followed by heating. All solvents used for HPLC were HPLC grade, while all solvents used for DFCC and TLC were at least of analytical grade.

### 3.2. Plant Material

The roots of *E. nicaeensis* All. (*Euphorbiaceae*) were collected from wild stock at Deliblato sands (Serbia, 44°56′57.4″ N, 21°11′13.5″ E) in May 2018. The plant was identified by prof. Petar Marin, Institute of Botany, Faculty of Biology, University of Belgrade. Voucher specimen (No. 16,855) has been deposited at the Herbarium of Botanical Garden “Jevremovac” University of Belgrade, Belgrade (Serbia).

### 3.3. Extraction and Isolation

The air-dried and grounded roots of *E. nicaeensis* were extracted by continuous extraction with 96% ethanol with heating under reflux for 2 h, and then it was left overnight at room temperature. The ethanolic extract (25 g) was subjected to silica-gel by dry-column flash chromatography (DCFC) [30] with petroleum ether–acetone (100:0, 90:10, 85:15, 80:20, 70:30) mixtures as eluents. The fraction F3 obtained using petroleum ether–acetone (85:15) (2.1 g) was separated further by DCFC on silica gel using a mixture of petroleum ether and acetone in different ratios (98:2, 97.5:2.5, 95:5, 90:10, 80:20) as a mobile phase. The collected fractions were monitored by TLC, and similar fractions were combined, giving thirteen fractions (1–13). The fraction F3/7 (178.7 mg) was subjected to the NP-HPLC on silica gel (Zorbax Rx-SIL column, 250 × 9.4 mm, 5 µm) with *n*-hexane and acetone (95:5, isocratic mode, flow 3 mL/min, 25 °C, 227 nm, stop time 30 min, post time 1 min) yielding fifteen subfractions, F3/7/I to F3/7/XV. The fraction F3/7/XII (4.0 mg) was finally purified using RP-HPLC (Zorbax XDB C18 column, 250 × 9.4 mm, 5 µm) with water and acetonitrile (ACN) in gradient mode as a mobile phase (50–80% ACN (0–10 min), 80–90% ACN (10–15 min), 90–100% ACN (15–21 min), flow 4 mL/min, 25 °C, 227 nm, stop time 21 min, post time 2 min), resulting in the isolation of compound **5** (1.9 mg). The fraction F3/10 (367.5 mg) was rechromatographed by NP-HPLC on silica gel (Zorbax Rx-SIL column, 250 × 9.4 mm, 5 µm) with *n*-hexane and acetone (95:5, isocratic mode, flow 3 mL/min, 25 °C, 227 nm, stop time 30 min, post time 1 min), yielding six subfractions, F3/10/I to F3/10/VI. The subfraction F3/10/I (3.6 mg) was finally subjected to the RP-HPLC (Zorbax XDB C18 column, 250 × 9.4 mm, 5 µm) with water and acetonitrile (ACN) in gradient mode as a mobile phase (50–80% ACN (0–10 min), 80–90% ACN (10–15 min), 90–100% ACN (15–21 min), flow 4 mL/min, 25 °C, 227 nm, stop time 21 min, post time 2 min), resulting in the isolation of compound **3** (0.7 mg). Subfractions F3/10/II (10.2 mg), F3/10/III (13.6 mg), and F3/10/V were further rechromatographed under the same conditions as fraction F3/10/I to give compounds **5** (4.0 mg), **4** (4.2 mg), and **2** (1.3 mg), respectively. The same RP-HPLC conditions were also used for final purifications of subfractions F3/10/IV (7.4 mg) and F3/10/VI (18.5 mg). Two compounds, **6** (2.7 mg) and **1** (0.6 mg) were isolated from the subfraction F3/10/IV. Compound **1** (9.7 mg) was also isolated from the subfraction F3/10/VI. Fraction F4, obtained using petroleum ether–acetone (80:20) (2.3 g), was chromatographed further by DCFC on silica gel using mixture of petroleum ether and acetone in different ratios (98:2, 97.5:2.5, 95:5, 90:10, 80:20) as a mobile phase. The collected fractions were monitored by TLC, and similar fractions were combined, giving eleven fractions (1–11). The subfraction F4/11 (786.5 mg) was rechromatographed by NP-HPLC on silica gel column (Zorbax Rx-SIL column, 250 × 9.4 mm, 5 µm) with *n*-hexane and acetone (95:5, isocratic mode, flow 3 mL/min, 25 °C, 227 nm, stop time 30 min, post time 1 min). This purification step provided ten fractions (F4/11/I to F4/11/X). Three of ten fraction were further purified on RP-HPLC using Zorbax XDB C18 column (250 × 9.4 mm, 5 µm) and a mixture of water and ACN in gradient mode as a mobile phase (50–80% ACN (0–10 min), 80–90% ACN (10–15 min), 90–100% ACN (15–21 min), flow 4 mL/min, 25 °C, 227 nm, stop time 21 min, post time 2 min). The subfraction F4/11/I (10.7 mg) gave **1** (1.3 mg), while subfraction F4/11/III (23.9 mg) gave **7** (0.8 mg) and the subfraction F4/11/X (2.3 mg) was a source of **8** (1.1 mg).

- *Nicaeenin H* (13α-Acetyloxy-12β-benzoyloxy-4-epi-4,20-dideoxyphorbol, **1**): colorless, amorphous, solid substance; [α]_D_^20^ -14.0 (c 0.10, MeOH); UV (MeOH) λ_max_ 195, 231 nm; IR (ATR) ν_max_ 2970, 1729, 1244, 1120, 1015 cm^−1^; ^1^H and ^13^C NMR data in Table 1 and Table 2; HRESIMS *m*/*z* 501.2246 [M + Na]^+^ (calcd for C_29_H_34_NaO_6_^+^ 501.2248).
- *Nicaeenin I* (13α-Acetyloxy-12β-benzoyloxy-4,20-dideoxyphorbol, **2**): colorless, amorphous, solid substance; [α]_D_^20^ +14.0 (c 0.10, MeOH); UV (MeOH) λ_max_ 201, 231 nm; IR (ATR) ν*max* 2981, 1730, 1228, 1124, 1025 cm^−1^; ^1^H and ^13^C NMR data in Table 1 and Table 2; HRESIMS *m*/*z* 479.2416 [M + H]^+^ (calcd for C_29_H_35_O_6_^+^ 479.2428).
- *Nicaeenin J* (12β-Benzoyloxy-13α-isovaleryloxy-4-epi-4,20-dideoxyphorbol, **3**): colorless, amorphous, solid substance; [α] _D_^20^ +9.9 (c 0.07, acetone); UV (MeOH) λ_max_ 201, 232 nm; IR (ATR) ν_max_ 2974, 1735, 1230, 1122, 1020 cm^−1^; ^1^H and ^13^C NMR data in Table 1 and Table 2; HRESIMS *m*/*z* 543.2714 [M + Na]+ (calcd for C_32_H_40_NaO_6_^+^ 543.2717).
- *Nicaeenin K* (12β-Acetyloxy-13α-isovaleryloxy-4-epi-4,20-dideoxyphorbol, **4**): colorless, amorphous, solid substance; [α] _D_^20^ +7.8 (c 0.28, acetone); UV (MeOH) λ_max_ 210, 236 nm; IR (ATR) ν_max_ 2969, 1731, 1233, 1129, 1022 cm^−1^; ^1^H and ^13^C NMR data data in Table 1 and Table 2; HRESIMS *m*/*z* 481.2542 [M + Na]^+^ (calcd for C_27_H_38_NaO_6_^+^ 481.2561).
- *Nicaeenin L* (13α,20-Diacetyloxy-12β-(2′E,4′E-nonadienoyloxy)-4-epi-deoxyphorbol, **7**): colorless, amorphous, solid; [α]_D_^20^ -1.3 (c 0.08, MeOH); UV (MeOH) λ_max_ 199, 268 nm; IR (ATR) ν_max_ 2974, 1733, 1235, 1126, 1024 cm^−1^; ^1^H and ^13^C NMR data in Table 1 and Table 2; HRESIMS *m*/*z* 583.3265 [M + H]+ (calcd for C_34_H_47_O_8_^+^ 583.3265).

### 3.4. Anti-HIV-Activity Investigation

The MT-4 cell line was a kind gift from Dr. L. Montagner (at that time at the Pasteur Institute; Paris, France). This cell line was cultured in RPMI medium (Thermo Fisher Scientific, Waltham, MA, USA) containing 10% fetal bovine serum (FBS; Thermo Fisher Scientific, Waltham, MA, USA) and 2 mM L-glutamine (Thermo Fisher Scientific, Waltham, MA, USA).

Freshly isolated peripheral blood mononuclear cells (PBMCs) were isolated out of buffy coats from healthy donors (Red Cross, Mechelen, Belgium). They were cultured in RPMI medium supplemented with 10% FBS, 2 mM L-glutamine, and 2 ng/mL interleukin-2 (IL-2, R&D Systems) and stimulated with 2 µg/mL phytohemagglutinin (PHA, Sigma-Aldrich, St. Louis, MO, USA) for three days before use in the HIV replication assays.

The HIV-1 strain NL4.3 (X4) and HIV-2 strain ROD (X4/R5) were obtained from the National Institute of Allergy and Infectious Disease AIDS program (Bethesda, MD, USA) and the Medical Research Council (MRC, London, UK), respectively. The HIV-1 strain HE (X4/R5) was originally isolated from a Belgian AIDS patient and was routinely cultured in MT-4 cells. The 50% tissue culture infectious doses of the virus stocks were used in the infection assays.

To determine the anti-HIV-activity of the compounds, MT-4 cells (5 × 10^4^ cells) were treated with 5-fold dilutions of the compounds for 30 min at 37 °C, 5% CO_2_ in 96-well-plates. After 30 min, cells were infected with 100 TCID50 (tissue culture infective dose 50%) HIV-1 (NL4.3) or HIV-2 (ROD) virus stocks. After five days of incubation (37 °C, 5% CO_2_), the cytopathic effect was checked microscopically, and cell viability was evaluated using the MTS/PES-based CellTiter 96 Aqueous One Solution Cell Proliferation assay (Promega Madison, WI, USA). Absorbance at 490 nm was measured using the VersaMax ELISATM microplate reader (Molecular Devices) and analyzed with the SoftMax Pro software (Molecular Devices, Version 4.0, www.moleculardevices.com, accessed on 10 January 2015). The IC_50_-values were calculated based on the absorbance signals measured in the negative (i.e., untreated and uninfected cells) and positive (i.e., untreated virus-infected cells) control samples.

PHA-activated PBMCs (5 × 10^5^ cells per sample) were pre-incubated (30 min at 37 °C, 5% CO_2_) with different concentrations of the compounds in cell culture medium containing 2 ng/mL IL-2 prior to infection with the HIV-1 NL4.3 and HE strains, and the HIV-2 ROD strain at a final dose of 100 TCID50. After four days, fresh culture medium with IL-2 was added. Cell supernatant was collected ten days post infection and viral replication was measured using a p24 HIV-1 Ag ELISA (Perkin Elmer, Waltham, MA, USA) according to the manufacturer’s guidelines.

The CC_50_ or 50% cellular cytoxic concentration of compounds was determined from the reduction of viability of uninfected MT-4 cells or PBMCs exposed to the compounds, as measured by the MTS method described above.

## 4. Conclusions

This chemical investigation of the *E. nicaeensis* root has revealed that the species is a rich source of biologically active compounds. It has been shown that different parts of the plant produce distinct classes of diterpenes. In the study, tigliane derivatives were isolated for the first time from *E. nicaeensis* root. Regarding the biological activities of the isolated compounds, none of them outperformed the reference drugs (PMPA, AMD3100) in terms of potency. However, compounds **2** and **8** demonstrated the best activity among all the isolated derivatives, with the activity of compound **8** being similar to that of PMPA but significantly weaker than AMD3100. Additionally, transesterification of the C-12 position with fatty acids could potentially lead to the formation of tigliane derivatives with significantly improved anti-HIV activity compared to the isolated molecules.

## Figures and Tables

**Table 1:** ^1^H NMR data of isolated compounds **1**–**4** and **7** (500 MHz, CDCl_3_, TMS, δ (ppm), *J* (Hz)).

	1	2	3	4	7
1	7.04, brs	7.58, brs	7.04, brs	6.99, brs	7.00, brs
4	2.71, m	2.49, m	2.71, m	2.68, m	2.75, m
5α	3.41, d, 16	2.86, dd, 18, 10	3.47, m	3.39, m	3.38, brd, 16
5β	2.38, dd, 16, 5	2.03, dd, 18, 10	2.38, dd, 15, 5	2.35, dd, 15, 5	2.47, dd, 16, 5
7	4.84, brs	5.24, d, 5	4.85, brs	4.81, brs	5.18, brs
8	1.97, brs	2.41, m	1.96, brs	1.88, brs	1.99, brs
9(OH)	5.13, s	5.55, s	5.24, s	5.13, s	5.17, brs
10	3.46, m	3.32, m	3.48, m	3.43, m	3.47, m
11	1.87, m	1.72, m	1.86, m	1.68, m	1.75, m
12	5.74, d, 10	5.67, d, 10	5.73, d, 11	5.43, d, 10	5.55, d, 10
14	0.87, d, 5	1.07, d, 5	0.84, d, 5	0.77, d, 5	0.82, d, 5
16	1.18, s	1.20, s	1.18, s	1.17, s	1.25, s
17	1.34, s	1.32, s	1.35, s	1.19, s	1.19, s
18	1.12, d, 6	0.97, d, 7	1.11, d, 7	1.06, d, 7	1.08, d, 6
19	1.80, s	1.73, brs	1.80, s	1.78, s	1.77, s
20	1.75, s	1.75, brs	1.76, s	1.74, s	4.47, d, 124.35, d, 12
12-OR					
2′				2.11, s	5.91, d, 15
3′	8.06, d, 7	8.02, d, 8	8.07, d, 8		7.66, dd, 15, 11
4′	7.48, t, 7	7.46, t,8	7.48, t, 8		6.17, t, 11
5′	7.60, t, 7	7.60, m	7.60, t, 8	-	5.92, m
6′	7.48, t, 7	7.46, t,8	7.48, t, 8	-	2.32, m
7′	8.06, d, 7	8.02, d, 8	8.07, d, 8	-	1.44, m
8′	-	-	-	-	1.33, m
9′	-	-	-	-	1.32, m
10′	-	-	-	-	0.91, t, 7
13-OR					
2″	2.09, s	2.13, s	2.21, m	2.18, m	2.13, s
3″	-	-	2.10, m	2.10, m	-
4″	-	-	0.97, d, 7	0.96, d, 7	-
5″	-	-	0.95, d, 7	0.94, d, 7	-
20-OR					
2‴	-	-	-	-	2.09, s

**Table 2:** ^13^C NMR data of isolated compounds **1**–**4** and **7** (125 MHz, CDCl_3_, TMS, δ (ppm)).

	1	2	3	4	7
1	155.5	160.2	155.7	155.7	155.4
2	143.2	136.7	143.4	143.3	143.6
3	211.7	210.2	212.0	211.9	211.2
4	49.2	44.8	49.5	49.4	49.1
5	30.0	34.3	30.2	30.1	26.6
6	134.9	139.3	135.1	134.9	133.0
7	124.2	125.9	124.5	124.4	129.0
8	40.9	42.5	41.2	41.0	41.2
9	78.1	78.2	78.3	78.1	78.0
10	47.1	54.6	47.3	47.2	47.1
11	43.3	42.8	43.8	43.4	43.4
12	76.7	78.1	77.0	76.3	75.7
13	65.4	65.7	65.3	65.2	65.5
14	37.7	36.2	38.1	37.8	37.1
15	25.2	25.6	25.7	25.6	25.4
16	24.2	24.0	24.5	24.4	16.6
17	16.6	17.2	16.9	16.5	24.3
18	11.9	15.4	12.2	12.1	12.1
19	10.5	10.4	10.7	10.6	10.7
20	29.7	26.0	29.2	29.0	70.4
12-OR					
1′	166.2	166.5	166.3	170.8	167.2
2′	130.1	130.2	130.5	21.2	120.8
3′	129.7	128.7	130.0	-	140.5
4′	128.5	129.9	128.7	-	126.6
5′	133.1	133.4	133.3	-	142.6
6′	128.5	129.9	128.7	-	28.5
7′	129.7	128.7	130.0	-	29.2
8′	-	-	-	-	31.6
9′	-	-	-	-	22.7
10′	-	-	-	-	14.2
13-OR					
1″	173.5	173.9	175.7	175.5	171.0
2″	21.1	21.4	43.7	43.6	21.3
3″	-	-	25.6	25.3	-
4″	-	-	22.7	22.5	-
5″	-	-	22.7	22.6	-
20-OR					
1‴	-	-	-	-	173.7
2‴	-	-	-	-	21.3

**Table 3:** IC_50_ (µM) and CC_50_ (µM) values of isolated compounds tested against HIV-1 NL4.3 and HIV-2 ROD replication in MT-4 cells.

	1	2	3	4	5	6	8	PMPA	AMD3100
HIV-1	>42.0 ± 0.0	7.5 ± 0.2	>18.0 ± 0.0	>52.0 ± 0.0	>34.0 ± 0.0	>101.0 ± 0.0	3.3 ± 1.8	2.4 ± 0.7	0.008 ± 0.002
HIV-2	9.4 ± 1.4	1.7 ± 1.3	>18.0 ± 0.0	>52.0 ± 0.0	4.6 ± 2.3	>101.0 ± 0.0	1.1 ± 0.8	0.7 ± 0.5	0.0075 ± 0.0005
Cellular toxicity	42.0 ± 0.0	94.0 ± 0.0	17.6 ± 0.3	52.0 ± 0.0	33.5 ± 0.4	101.0 ± 0.0	20.8 ± 5.6	>100.0 ± 0.0	>10.0 ± 0.0

## Data Availability

All data created during this study are presented in the manuscript or in the Supplementary Materials.

## Supplementary Materials

The following supporting information can be downloaded at https://www.mdpi.com/article/10.3390/molecules30071452/s1: Figure S1: ^1^H-NMR (500 MHz, CDCl_3_) spectrum of compound **1**; Figure S2: ^13^C-NMR (125 MHz, CDCl_3_) spectrum of compound **1**, Figure S3: HSQC spectrum of compound **1**, Figure S4: COSY spectrum of compound **1**, Figure S5: HMBC spectrum of compound **1**, Figure S6: NOESY spectrum of compound **1**, Figure S7: Mass spectrum of compound **1**, Figure S8: ^1^H-NMR (500 MHz, CDCl_3_) spectrum of compound **2**, Figure S9: ^13^C-NMR (125 MHz, CDCl_3_) spectrum of compound **2**, Figure S10: HSQC spectrum of compound **2**, Figure S11: COSY spectrum of compound **2**, Figure S12: HMBC spectrum of compound **2**, Figure S13: NOESY spectrum of compound **2**, Figure S14: Mass spectrum of compound **2**, Figure S15: ^1^H-NMR (500 MHz, CDCl_3_) spectrum of compound **3**, Figure S16: ^13^C-NMR (125 MHz, CDCl_3_) spectrum of compound **3**, Figure S17: HSQC spectrum of compound **3**, Figure S18: COSY spectrum of compound **3**, Figure S19: HMBC spectrum of compound **3**, Figure S20: NOESY spectrum of compound **3**, Figure S21: Mass spectrum of compound **3**, Figure S22: ^1^H-NMR (500 MHz, CDCl_3_) spectrum of compound **4**, Figure S23: ^13^C-NMR (125 MHz, CDCl_3_) spectrum of compound **4**, Figure S24: HSQC spectrum of compound **4**, Figure S25: COSY spectrum of compound **4**, Figure S26: HMBC spectrum of compound **4**, Figure S27: NOESY spectrum of compound **4**, Figure S28: Mass spectrum of compound **4,** Figure S29: ^1^H-NMR (500 MHz, CDCl_3_) spectrum of compound **5**, Figure S30: ^13^C-NMR (125 MHz, CDCl_3_) spectrum of compound **5**, Figure S31: HSQC spectrum of compound **5**, Figure S32: COSY spectrum of compound **5**, Figure S33: HMBC spectrum of compound **5**, Figure S34: Mass spectrum of compound **5**, Figure S35: ^1^H-NMR (500 MHz, CDCl_3_) spectrum of compound **6**, Figure S36: ^13^C-NMR (125 MHz, CDCl_3_) spectrum of compound **6**, Figure S37: HSQC spectrum of compound **6**, Figure S38: COSY spectrum of compound **6**, Figure S39: HMBC spectrum of compound **6**, Figure S40: NOESY spectrum of compound **6**, Figure S41: Mass spectrum of compound **6**, Figure S42: ^1^H-NMR (500 MHz, CDCl_3_) spectrum of compound **7**, Figure S43: ^13^C-NMR (125 MHz, CDCl_3_) spectrum of compound **7**, Figure S44: HSQC spectrum of compound **7**, Figure S45: COSY spectrum of compound **7**, Figure S46: HMBC spectrum of compound **7**, Figure S47: NOESY spectrum of compound **7**, Figure S48: Mass spectrum of compound **7**, Figure S49: ^1^H-NMR (500 MHz, CDCl_3_) spectrum of compound **8**, Figure S50: ^13^C-NMR (125 MHz, CDCl_3_) spectrum of compound **8**, Figure S51: HSQC spectrum of compound **8**, Figure S52: COSY spectrum of compound **8**, Figure S53: HMBC spectrum of compound **8**, and Figure S54: NOESY spectrum of compound **8**, Figure S55: Mass spectrum of compound **8**.
