# Supplementary material for: Anti-HIV Activity of Tigliane Derivatives from Euphorbia nicaeensis Roots
Source: Molecules. 2025 Mar 25;30(7):1452. doi: 10.3390/molecules30071452 (PMC11990108; doi:10.3390/molecules30071452)

Anti-HIV Activity of Tigliane Derivatives from *Euphorbia nicaeensis* Roots

Gordana Krstić[1,*], Milka Jadranin[2], Dominique Schols[3], Sandra Claes[3], Vele Tešević[1], Boris Mandić[1], Slobodan Milosavljević[1,4] and Karlo Wittine[5,*]

1 University of Belgrade – Faculty of Chemistry, Studentski trg 12–16, Belgrade 11010, Serbia (gkrstic@chem.bg.ac.rs (G.K.), vtesevic@chem.bg.ac.rs (V.T.), borism@chem.bg.ac.rs (B.M.), smilo@chem.bg.ac.rs (S.M.))
2 University of Belgrade – Institute of Chemistry, Technology and Metallurgy, Department of Chemistry, Njegoševa 12, Belgrade 11000, Serbia (milka.jadranin@ihtm.bg.ac.rs)
3 Laboratory of Molecular, Structural and Translational Virology, Rega Institute Herestraat 49, 3000 Leuven, Belgium (dominique.schols@kuleuven.be (D.S), sandra.claes@kuleuven.be (S.C.))
4 Serbian Academy of Science and Arts, Kneza Mihaila 35, Belgrade 11000, Serbia
5 Selvita Ltd., Zagreb, Prilaz baruna Filipovića 29, 10000, Zagreb, Croatia (karlo.wittine@selvita.com (K.W.))
6 Faculty of Biotechnology and Drug Development, University of Rijeka, Radmile Matejčić 2, 51000 Rijeka, Croatia (karlo.wittine@biotech.uniri.hr (K.W.))
* Correspondence: gkrstic@chem.bg.ac.rs (G.K.) and karlo.wittine@selvita.com (K.W.)

Figure S1: ^{1}H-NMR (500 MHz, $CDCl_3$) spectrum of compound **1**

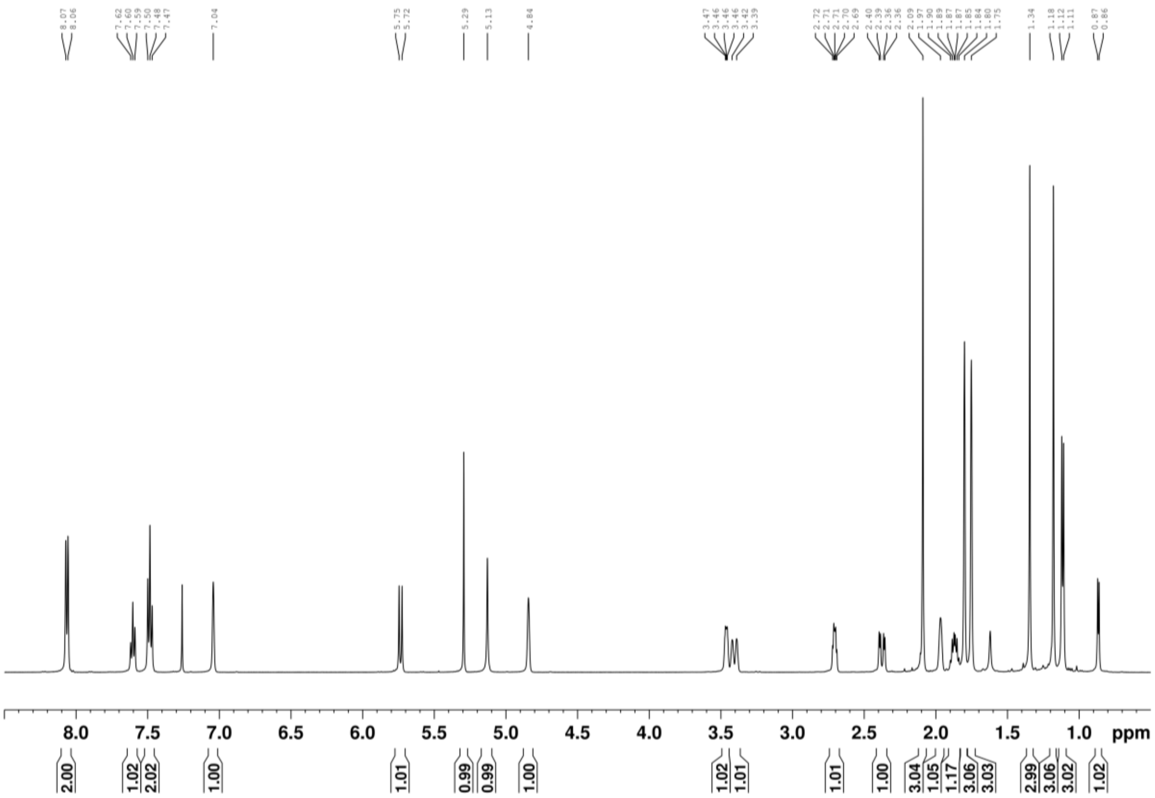

Figure S2: ^{13}C-NMR (125 MHz, $CDCl_3$) spectrum of compound **1**

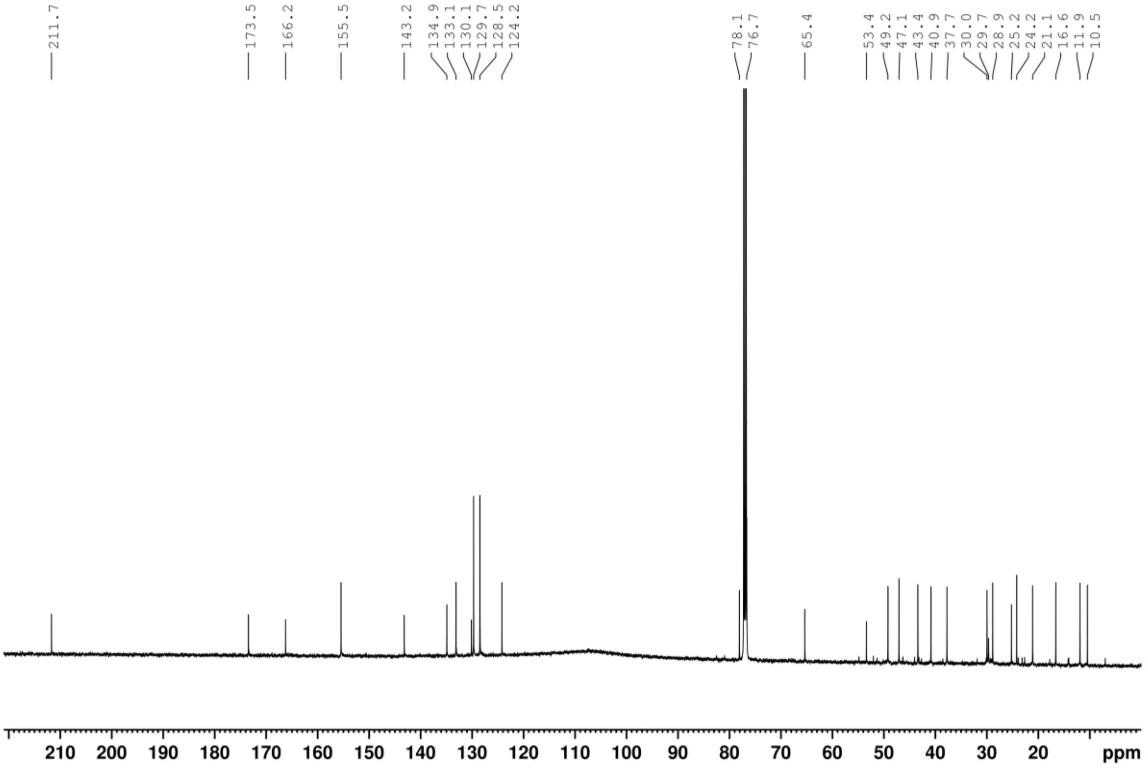

Figure S3: HSQC spectrum of compound **1**

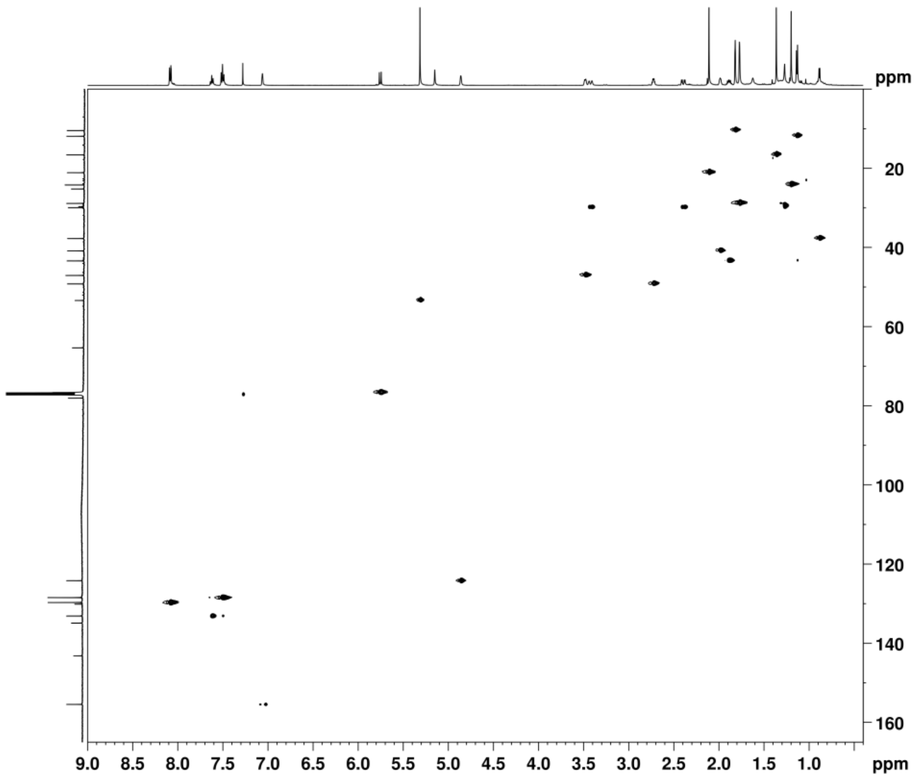

Figure S4: COSY spectrum of compound **1**

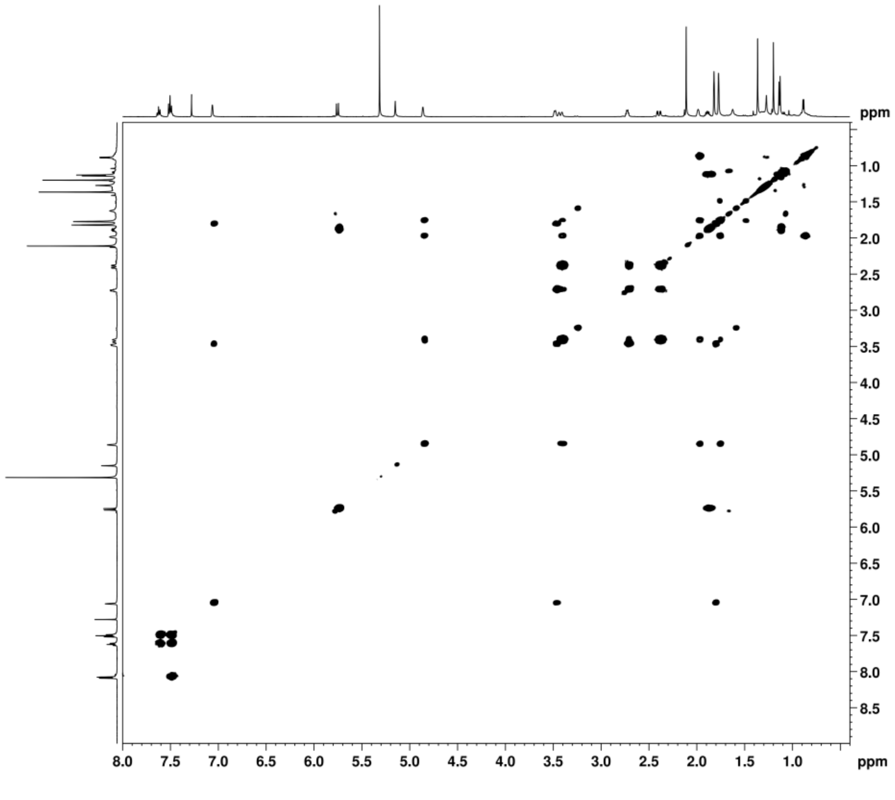

Figure S5: HMBC spectrum of compound **1**

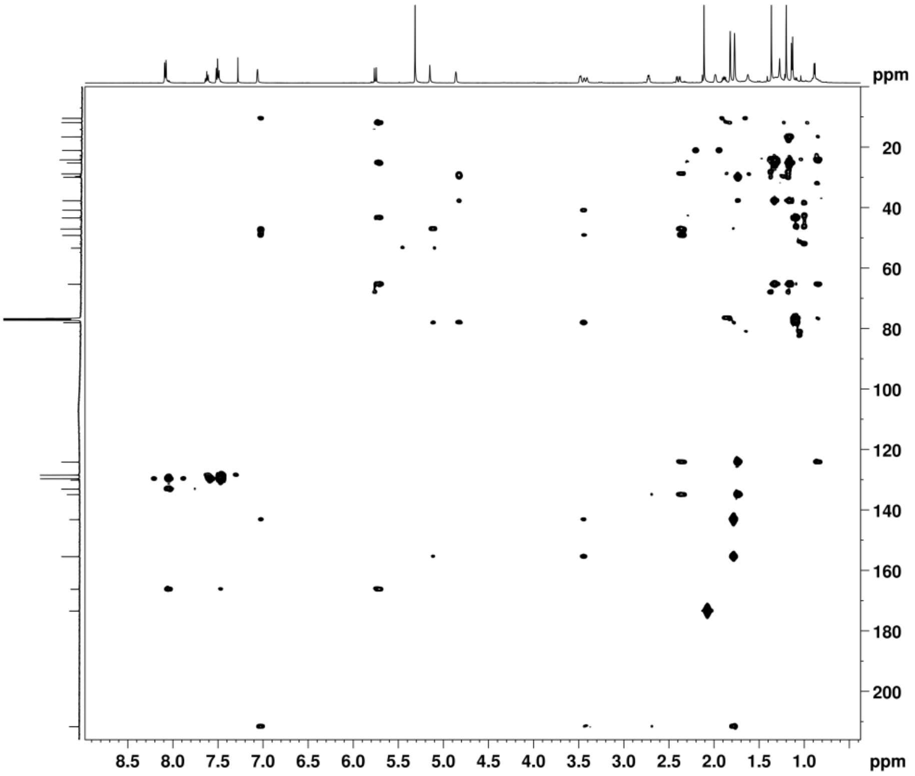

Figure S6: NOESY spectrum of compound **1**

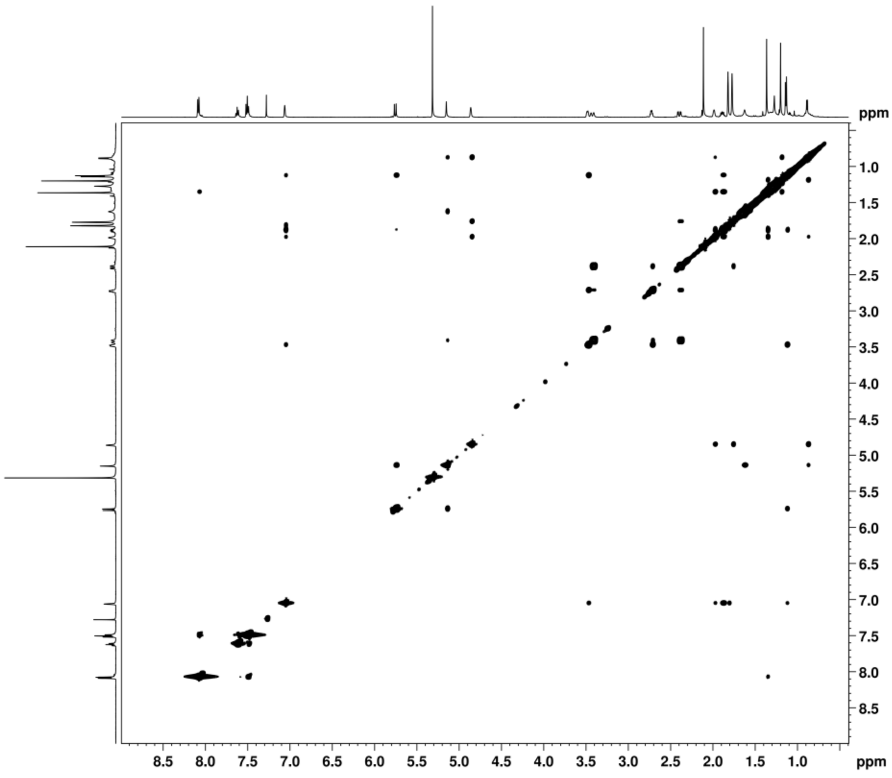

Figure S7: Mass spectrum of compound **1**

Qualitative Compound Report

**Data File**	GK_EGK-3-10-6-1_MK_70V_pos1.d	**Sample Name**	Unavailable
**Sample Type**	Unavailable	**Position**	Unavailable
**Instrument Name**	Unavailable	**User Name**	Unavailable
**Acq Method**		**Acquired Time**	Unavailable
**IRM Calibration Status**	Success	**DA Method**	Default.m
**Comment**	Sample information is unavailable		

jcd. 40

Compound Table

Compound Label	RT	Mass	Abund	Formula	Tgt Mass	Diff (ppm)
Cpd 1: C29 H34 O6	0.382	478.2352	9919	C29 H34 O6	478.2355	-0.71

Compound Label	*m/z*	RT	Algorithm	Mass
Cpd 1: C29 H34 O6	501.2246	0.382	Find By Formula	478.2352

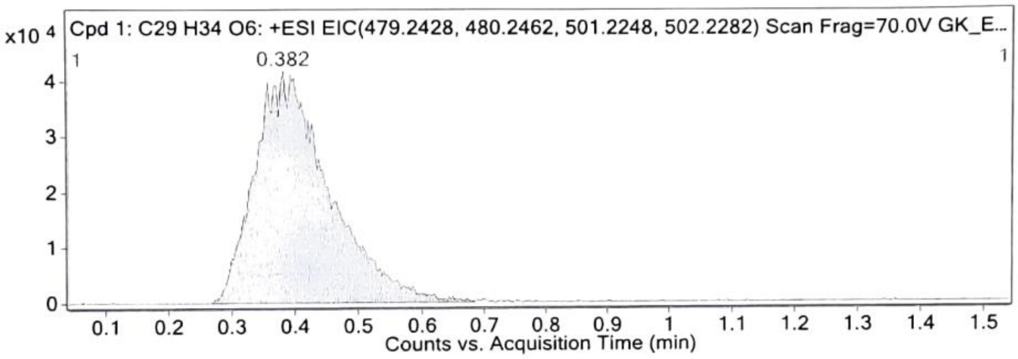

Figure S8: ^{1}H-NMR (500 MHz, $CDCl_3$) spectrum of compound **2**

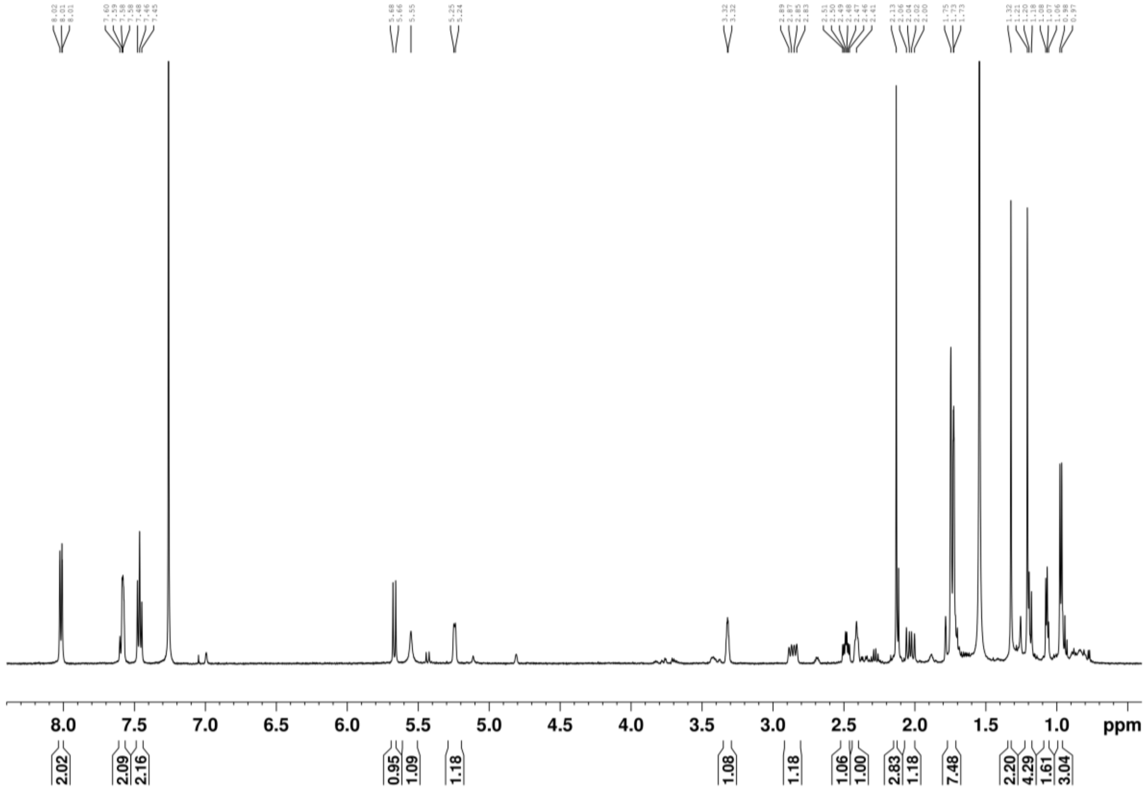

Figure S9: ^{13}C-NMR (125 MHz, $CDCl_3$) spectrum of compound **2**

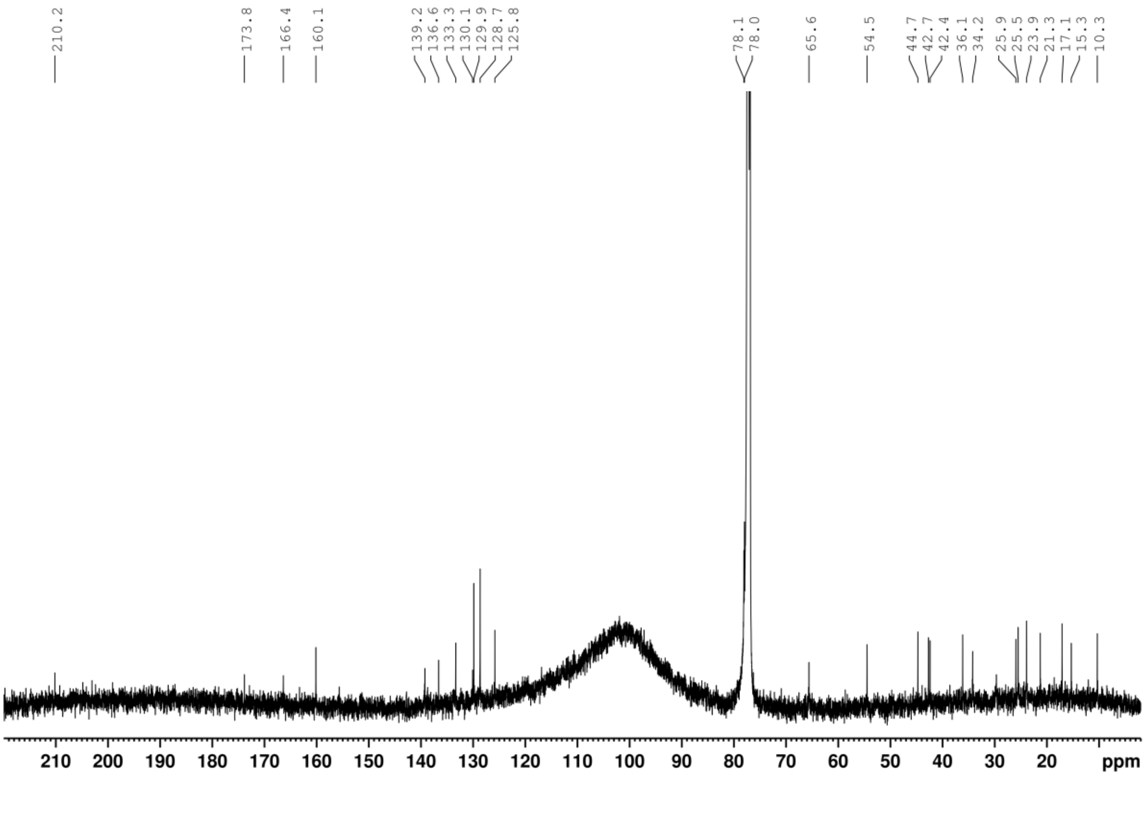

Figure S10: HSQC spectrum of compound **2**

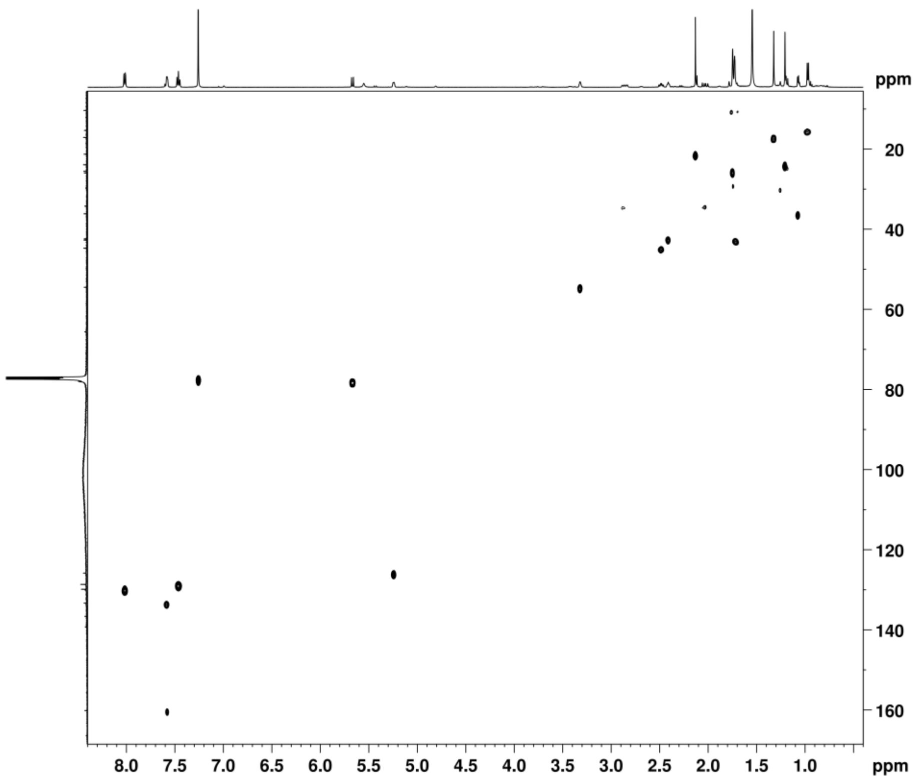

Figure S11: COSY spectrum of compound **2**

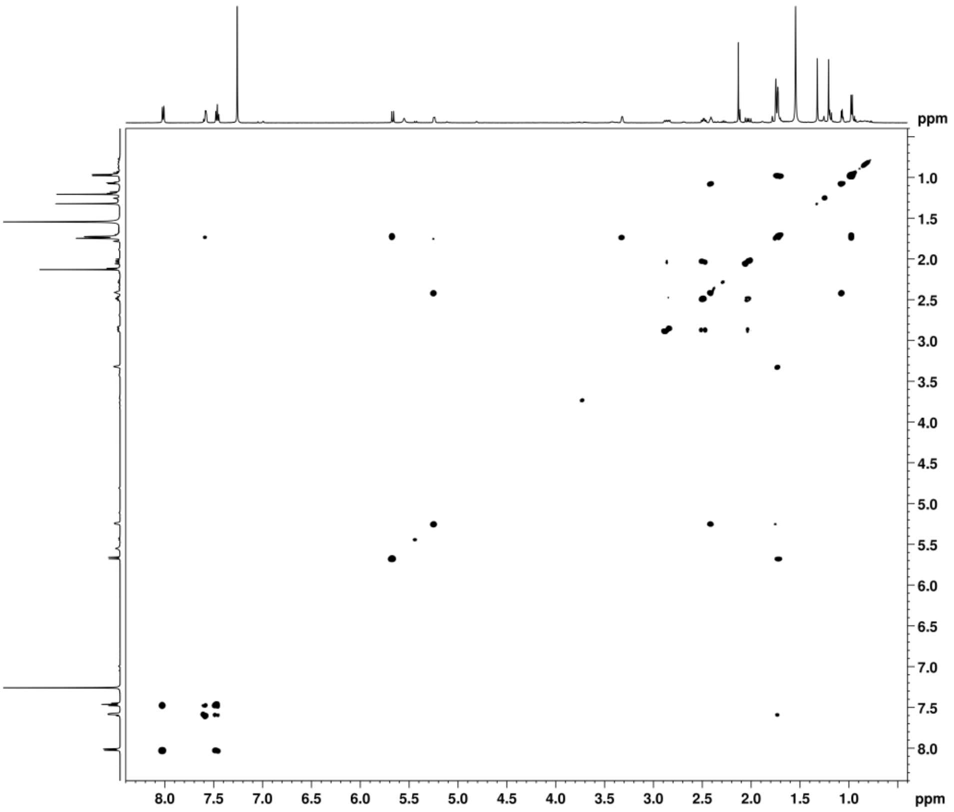

Figure S12: HMBC spectrum of compound **2**

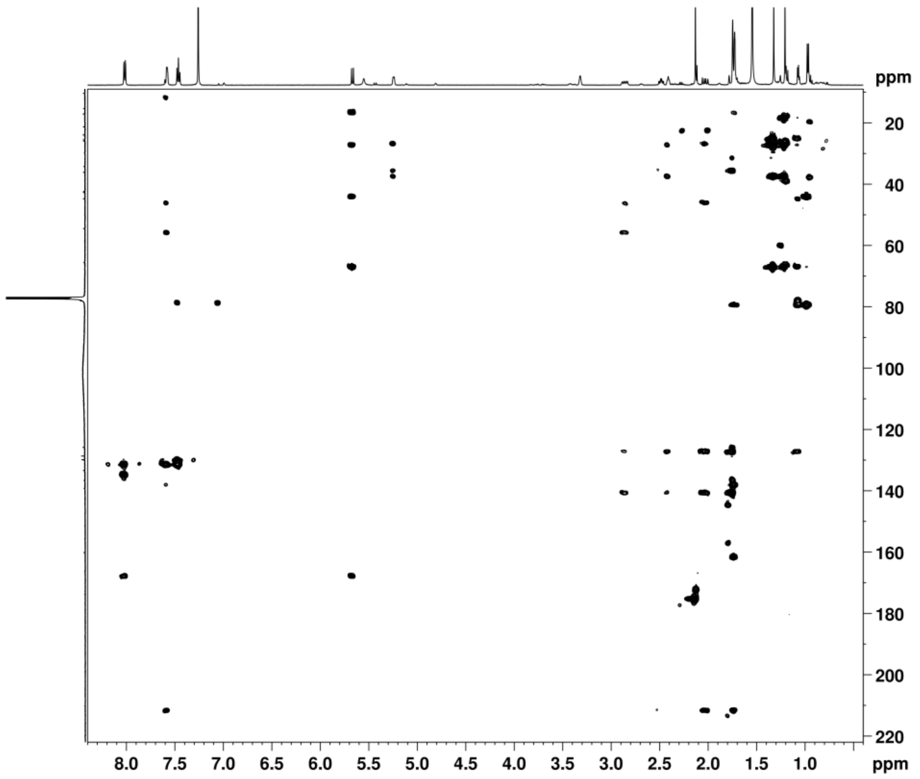

Figure S13: NOESY spectrum of compound **2**

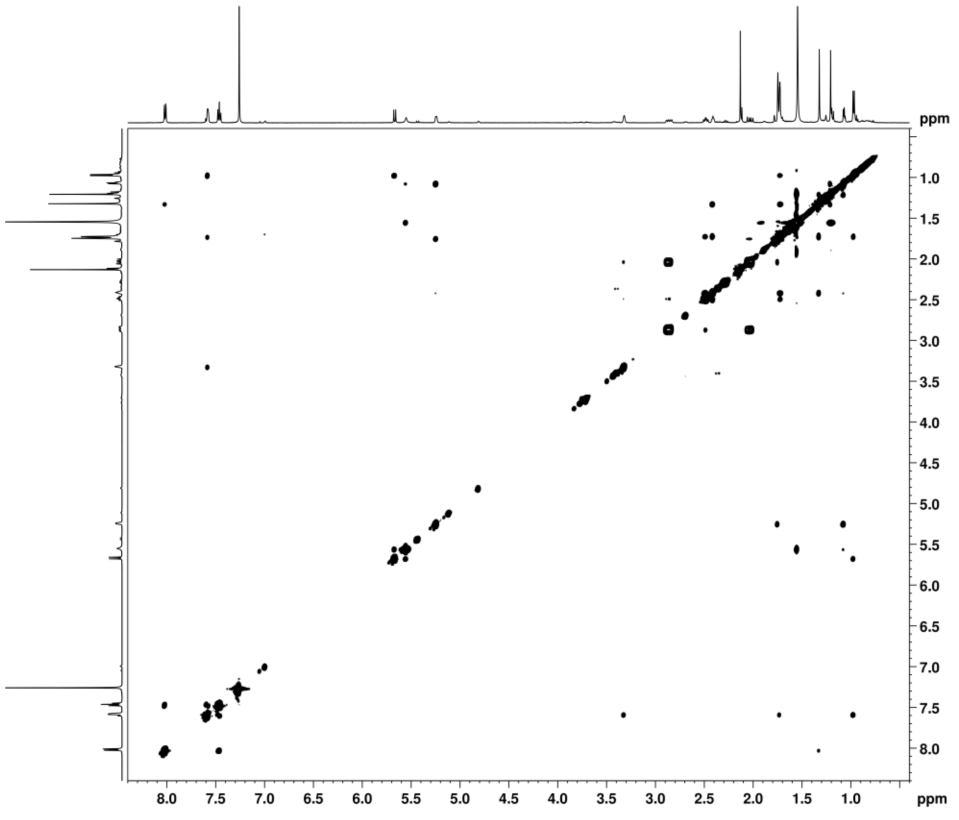

Figure S14: Mass spectrum of compound **2**

Qualitative Compound Identification Report

**Data File**	GK_EGK-3-10-5-3_ZEP_C18_10cm_MK_140V_pos1.d	**Sample Name**	EGK-3-10-5-3
**Sample Type**	Sample	**Position**	P1-C3
**Instrument Name**	DE1517B001	**User Name**	
**Acq Method**	Odredjivanje MM_ZEP_C18_10cm_mK_140V_pos.m	**Acquired Time**	31-Oct-19 10:54:43 (UTC+01:00)
**IRM Calibration Status**	Some Ions Missed	**DA Method**	Lipidomics_qualan.m
**Comment**			
**Sample Group**		**Info.**	
**Stream Name**	LC 1	**Acquisition Time (Local)**	31-Oct-19 10:54:43 (UTC+01:00)
**Acquisition SW Version**	6200 series TOF/6500 series Q-TOF B.06.01 (B6157)	**QTOF Driver Version**	6.00.01
**QTOF Firmware Version**	14.643	**Tune Mass Range Max.**	3200

Compound Table

Compound Label	RT	Mass	Abund	Formula	Tgt Mass	Diff (ppm)	Hits (DB)
Cpd 1: C29 H34 O6; 12.285	12.285	478.2343	10633	C29 H34 O6	478.2355	-2.54	1

Compound Label	*m/z*	RT	Algorithm	Mass
Cpd 1: C29 H34 O6; 12.285	479.2416	12.285	Find by Formula	478.2343

Compound Chromatograms

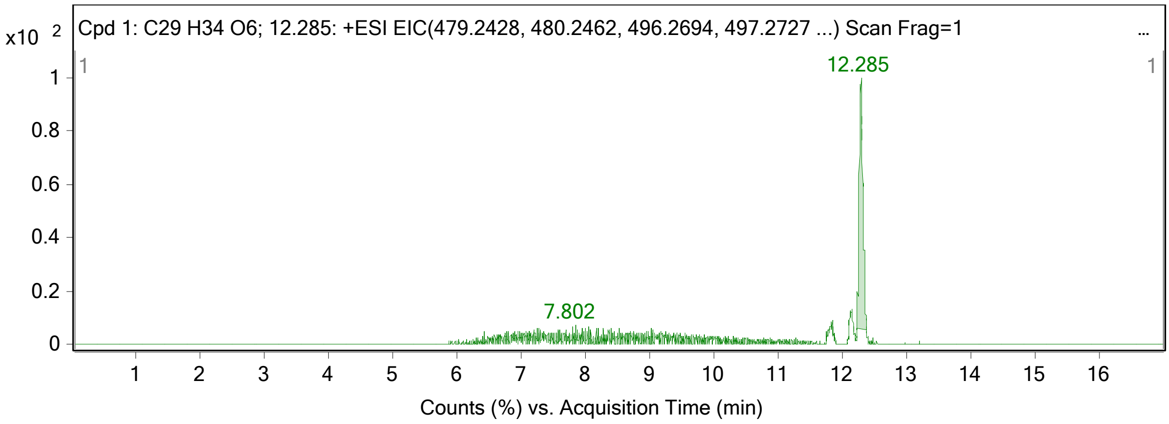

Figure S15: ^{1}H-NMR (500 MHz, $CDCl_3$) spectrum of compound **3**

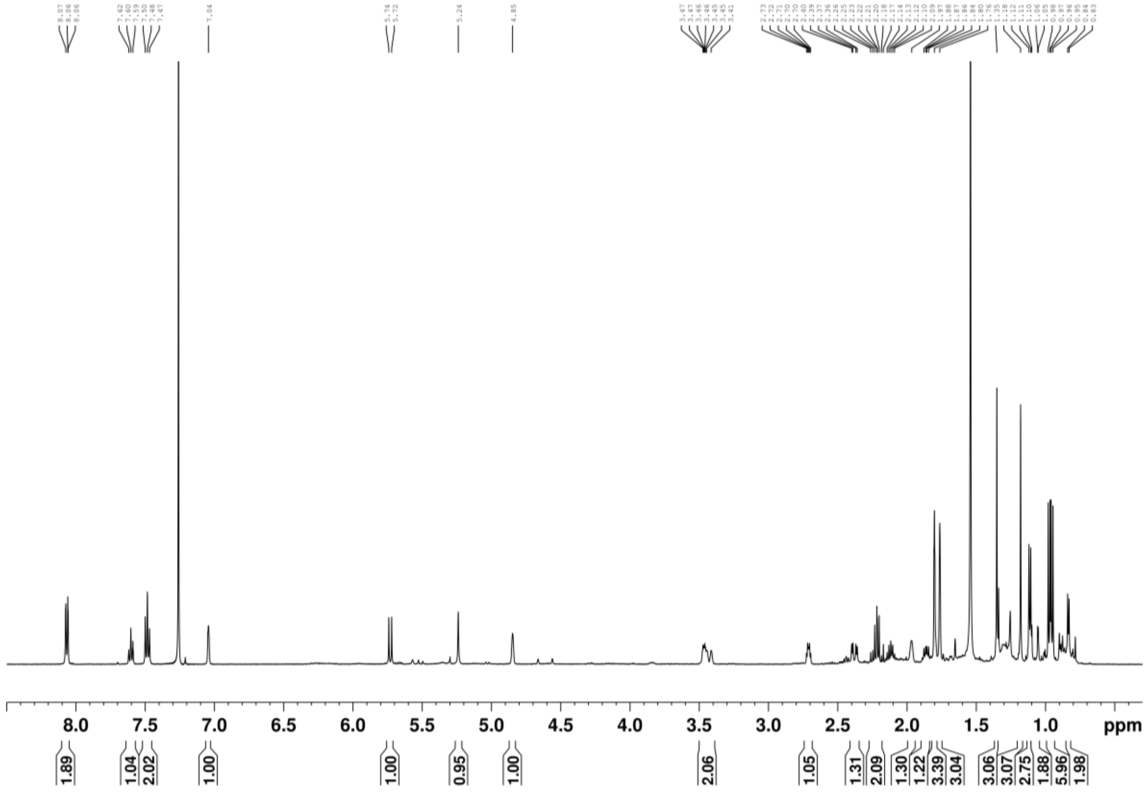

Figure S16: ^{13}C-NMR (125 MHz, $CDCl_3$) spectrum of compound **3**

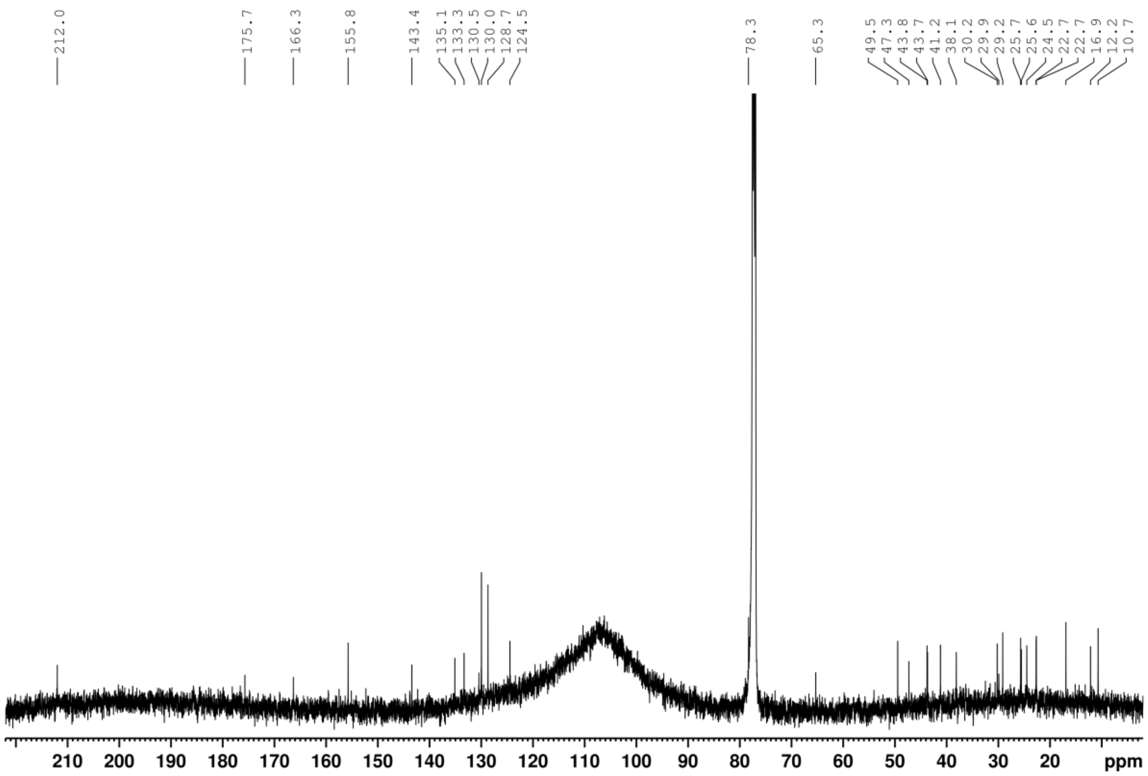

Figure S17: HSQC spectrum of compound **3**

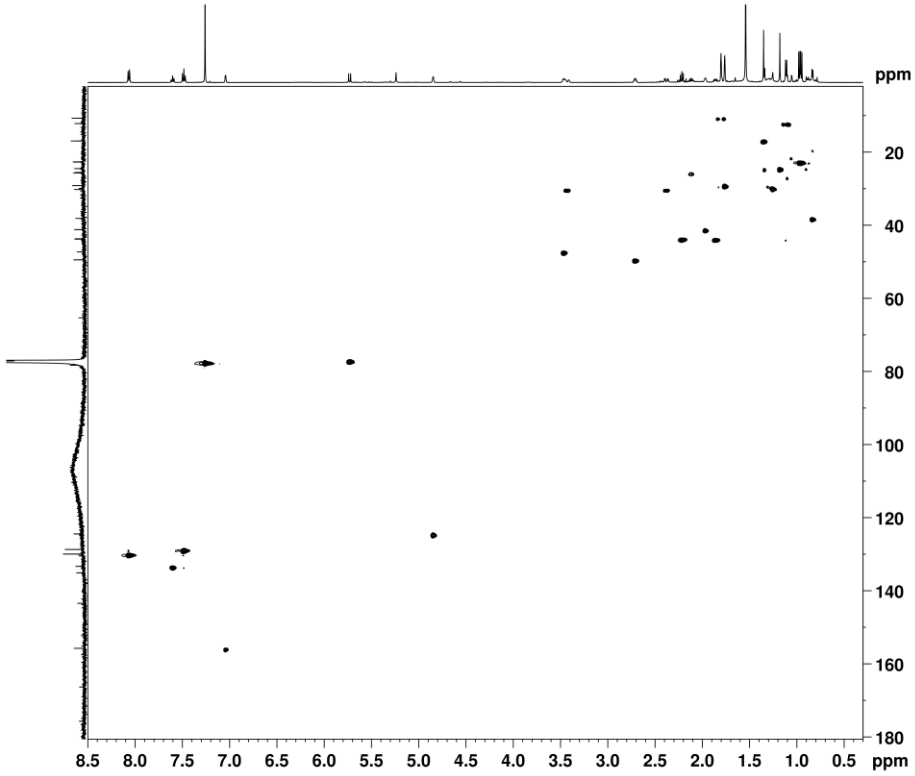

Figure S18: COSY spectrum of compound **3**

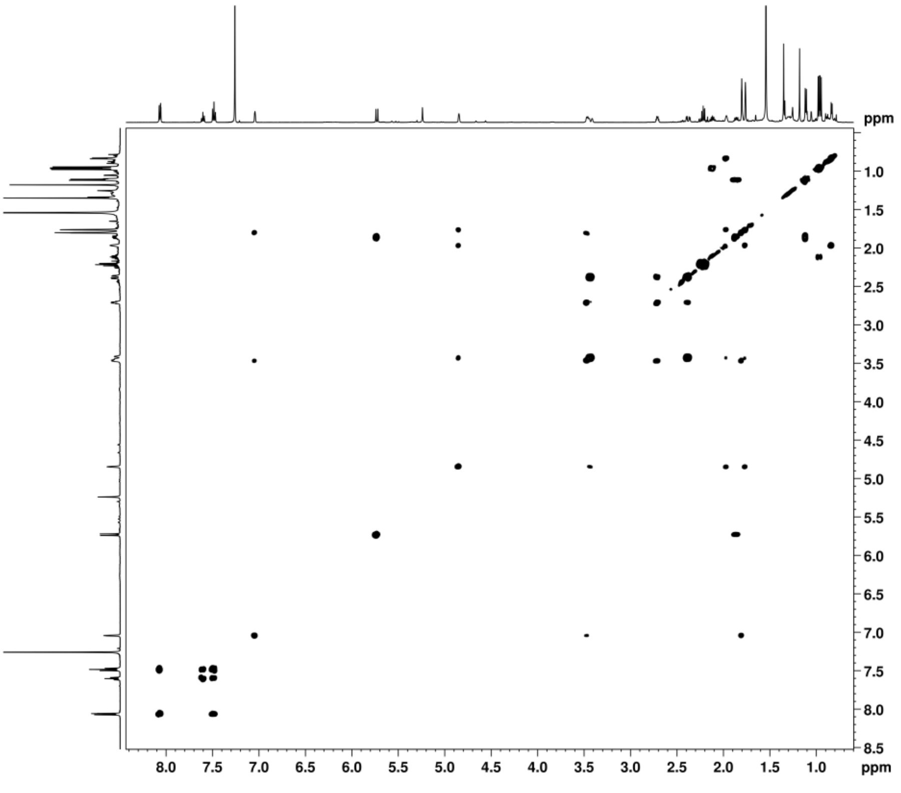

Figure S19: HMBC spectrum of compound **3**

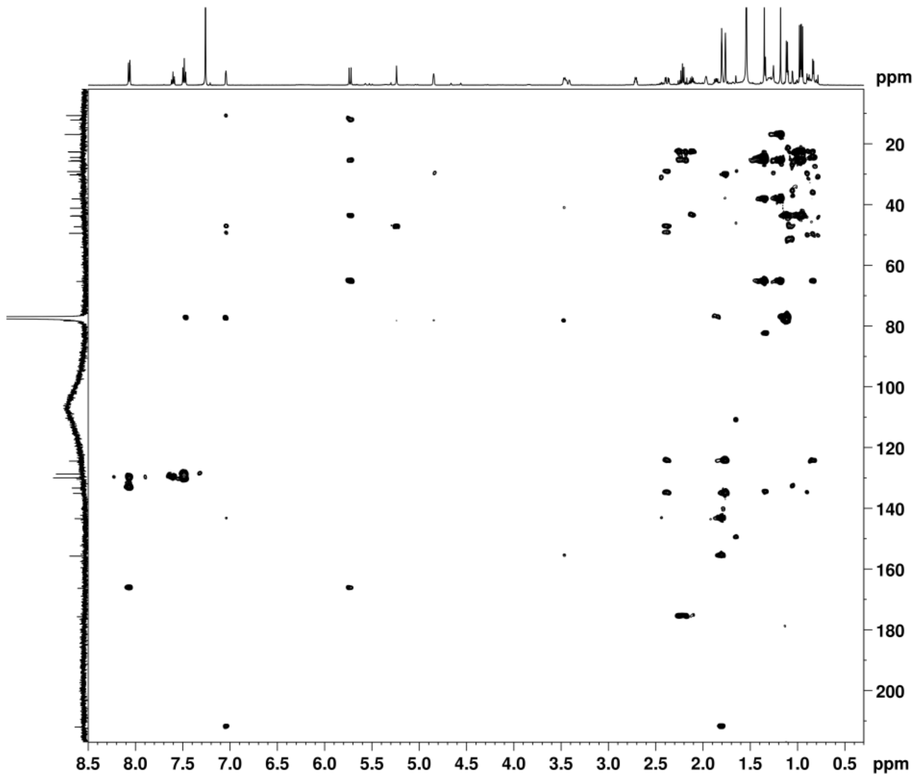

Figure S20: NOESY spectrum of compound **3**

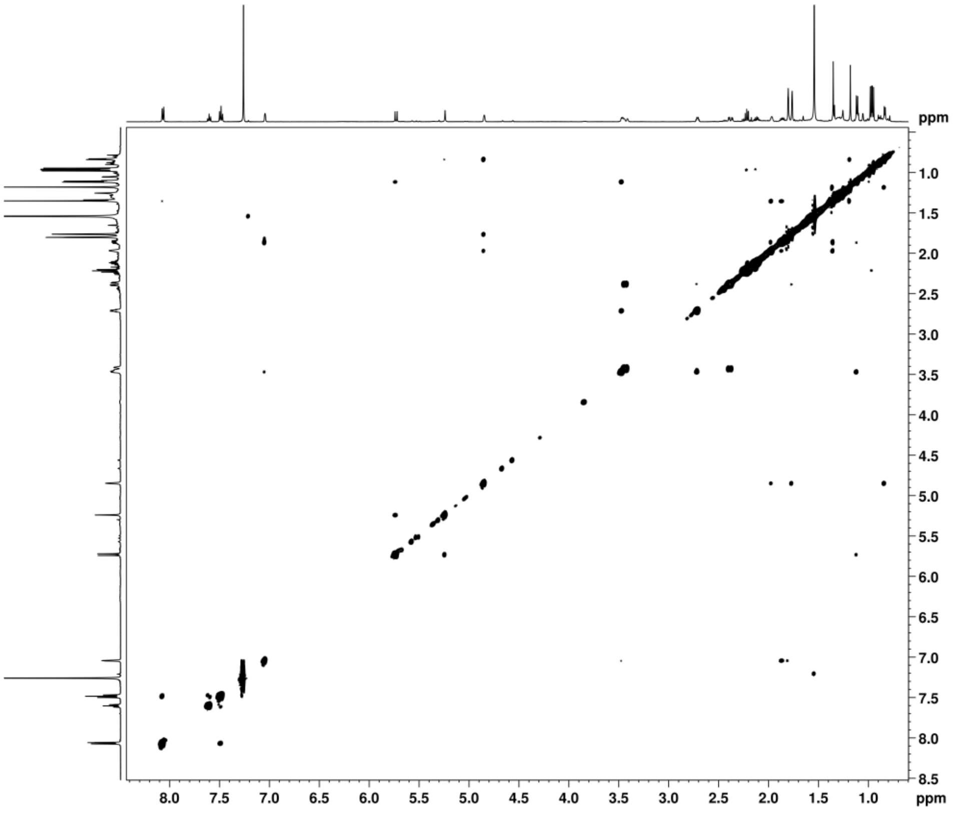

Figure S21: Mass spectrum of compound **3**

Qualitative Compound Report

**Data File**	GK_EGK-3-10-1-1_MK_70V_pos1.d	**Sample Name**	Unavailable
**Sample Type**	Unavailable	**Position**	Unavailable
**Instrument Name**	Unavailable	**User Name**	Unavailable
**Acq Method**		**Acquired Time**	Unavailable
**IRM Calibration Status**	Success	**DA Method**	Default.m
**Comment**	Sample information is unavailable		

Compound Table

Compound Label	RT	Mass	Abund	Formula	Tgt Mass	Diff (ppm)
Cpd 1: C32 H40 O6	0.382	520.2821	7625	C32 H40 O6	520.2825	-0.83

Compound Label	*m/z*	RT	Algorithm	Mass
Cpd 1: C32 H40 O6	543.2714	0.382	Find By Formula	520.2821

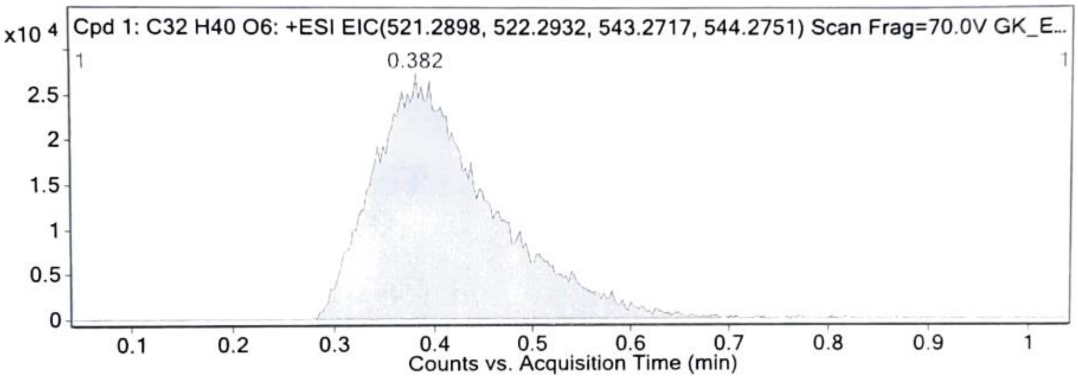

Figure S22: ^{1}H-NMR (500 MHz, $CDCl_3$) spectrum of compound **4**

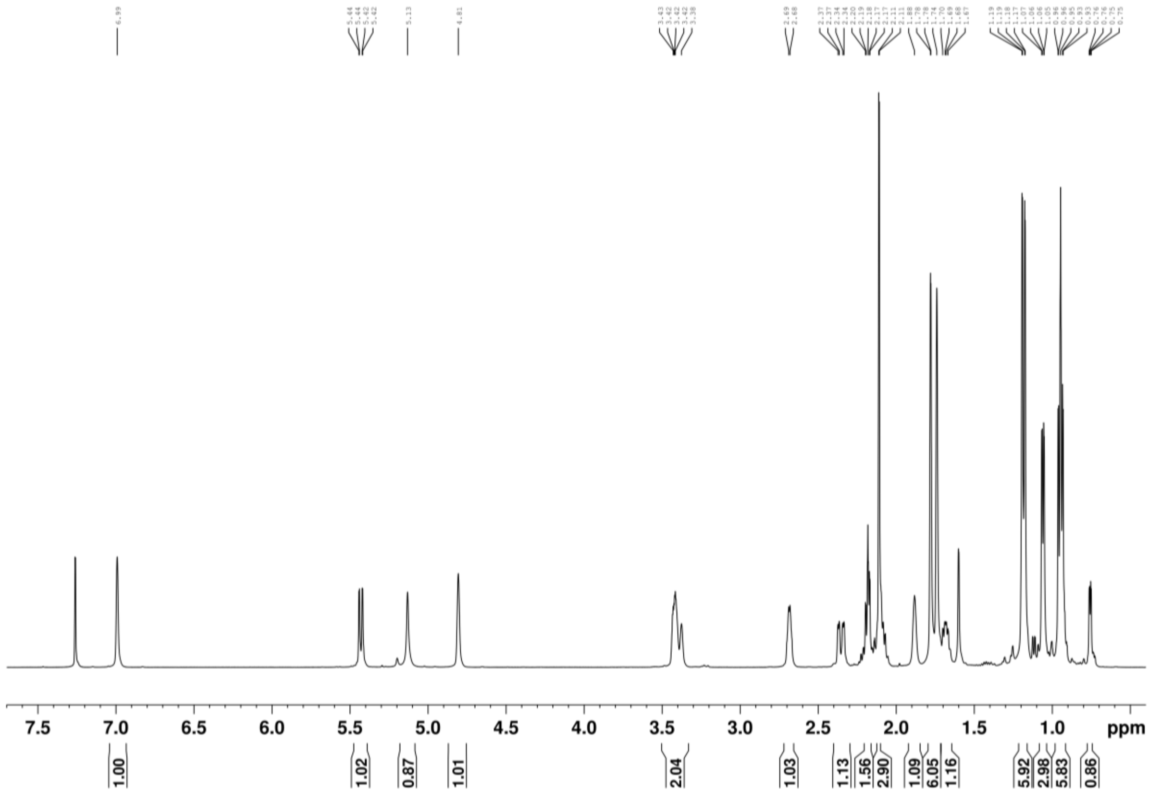

Figure S23: ^{13}C-NMR (125 MHz, $CDCl_3$) spectrum of compound **4**

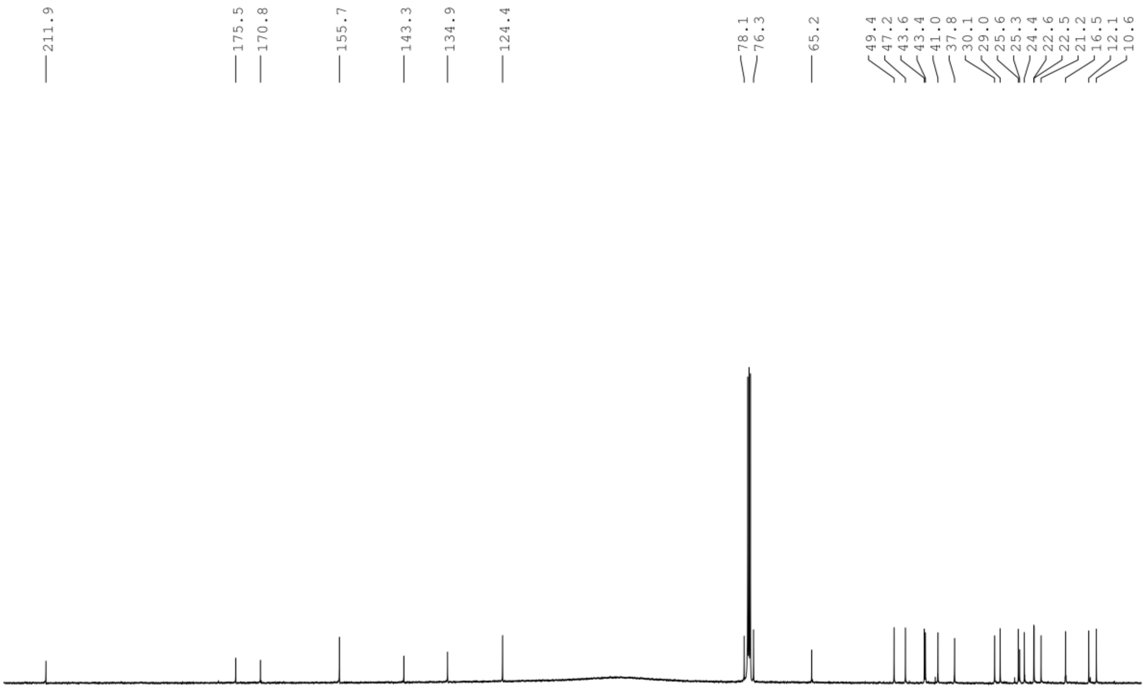

Figure S24: HSQC spectrum of compound **4**

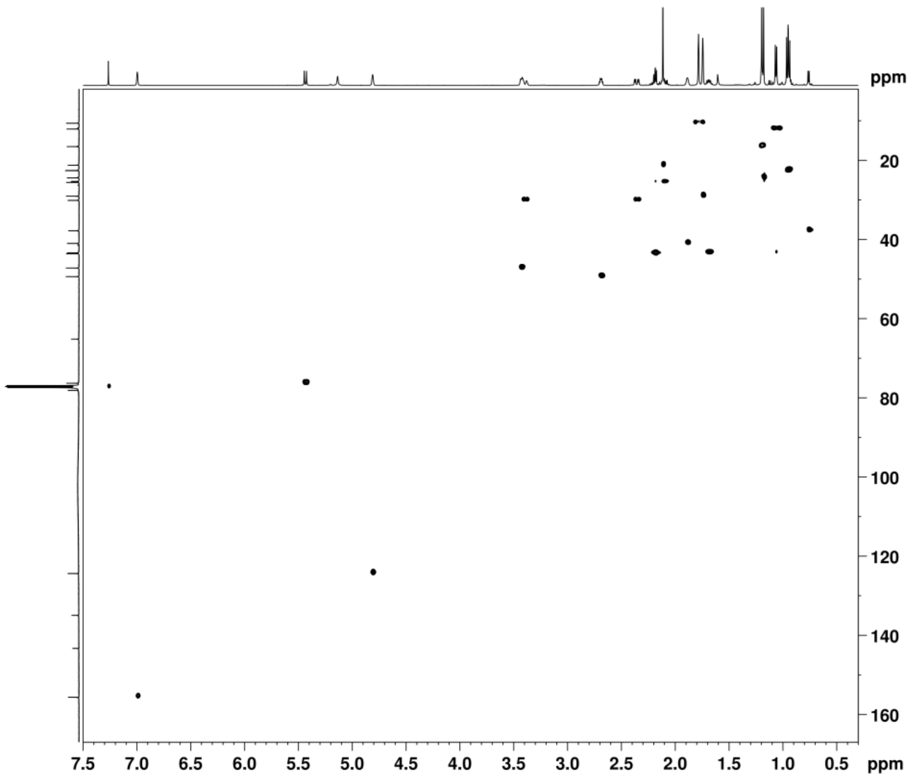

Figure S25: COSY spectrum of compound **4**

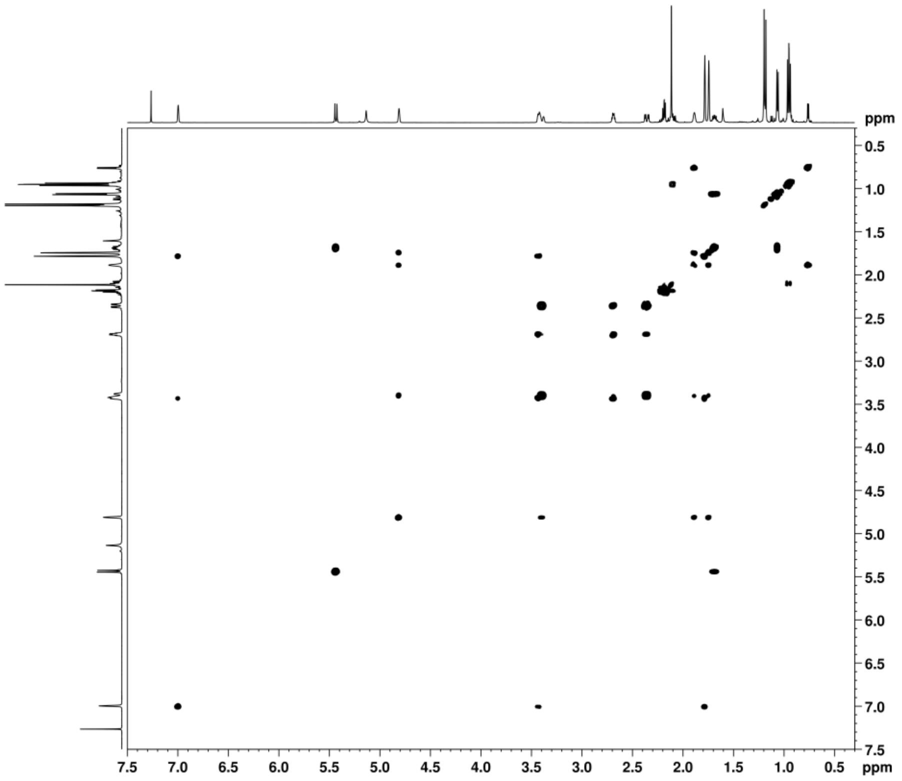

Figure S26: HMBC spectrum of compound **4**

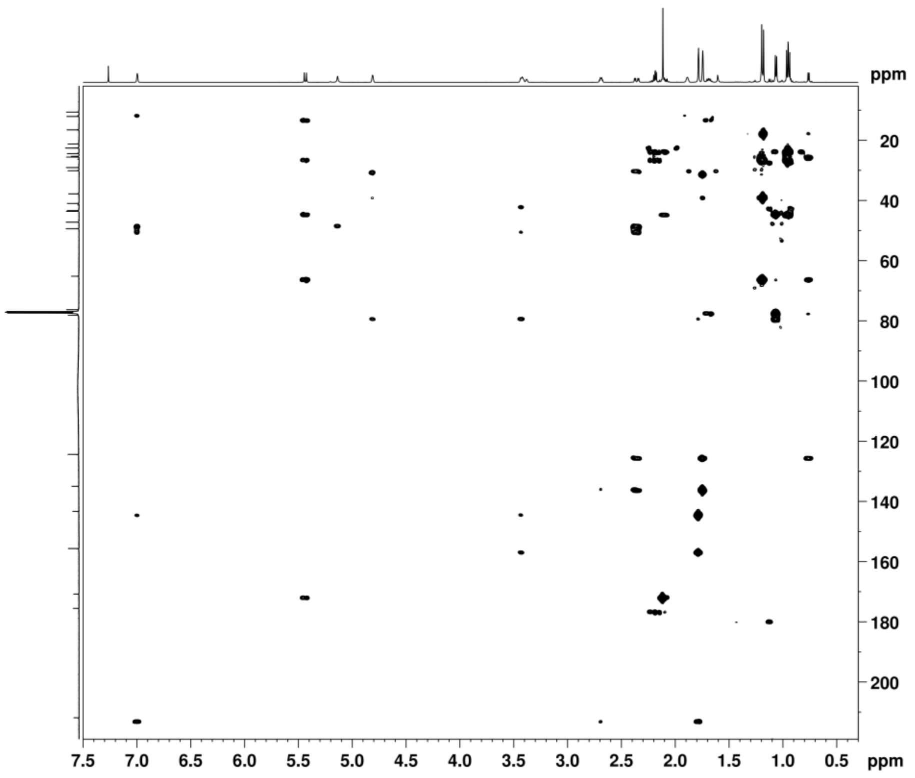

Figure S27: NOESY spectrum of compound **4**

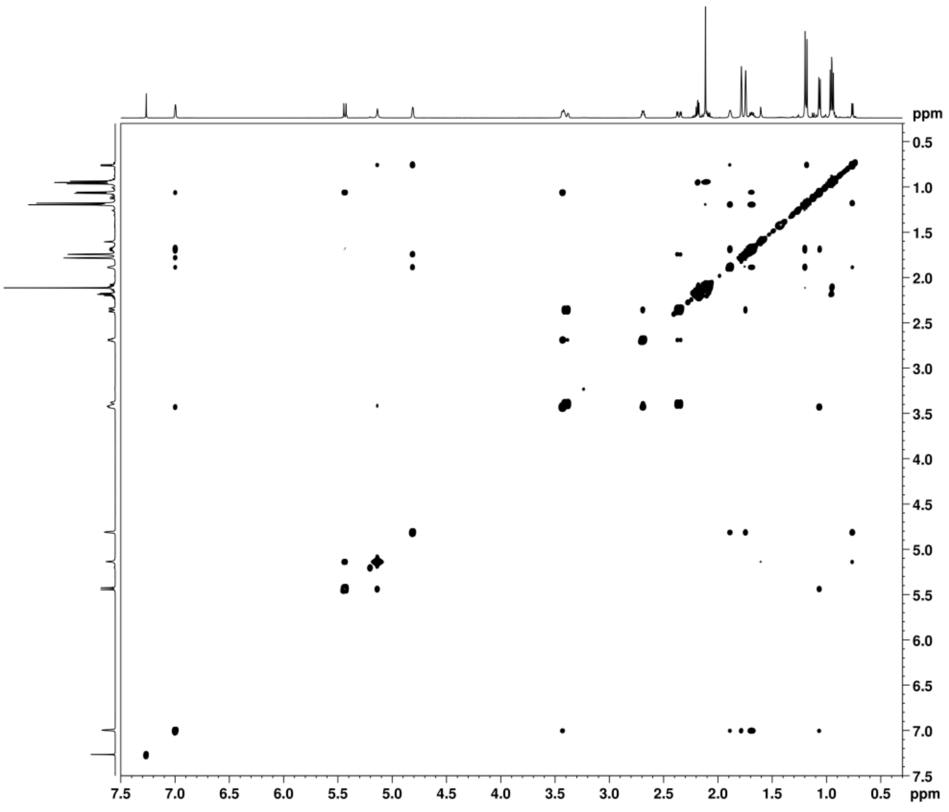

Figure S28: Mass spectrum of compound **4**

Qualitative Compound Report

**Data File**	GK_EGK-3-10-3-1_ZEP_C18_10cm_MK_140V_pos1.d	**Sample Name**	EGK-3-10-3-1
**Sample Type**	Sample	**Position**	P1-C4
**Instrument Name**	DE1517B001	**User Name**	
**Acq Method**	Odredjivanje MM_ZEP_C18_10cm_mK_140V_pos.m	**Acquired Time**	10/31/2019 11:15:35 AM
**IRM Calibration Status**	Success	**DA Method**	Odredjivanje MM_ZEP_C18_10cm_mK_140V_pos.m
**Comment**			

**Sample Group**		**Info.**	
**Stream Name**	LC 1	**Acquisition SW Version**	6200 series TOF/6500 series Q-TOF B.06.01 (B6157)

Compound Table

Compound Label	RT	Mass	Abund	Formula	Tgt Mass	Diff (ppm)
Cpd 1: C27 H38 O6	11.714	458.2652	1842	C27 H38 O6	458.2668	-3.58

Compound Label	*m/z*	RT	Algorithm	Mass
Cpd 1: C27 H38 O6	481.2542	11.714	Find By Formula	458.2652

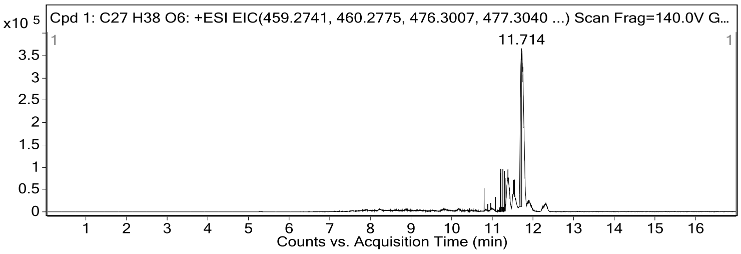

Figure S29: ^{1}H-NMR (500 MHz, $CDCl_3$) spectrum of compound **5**

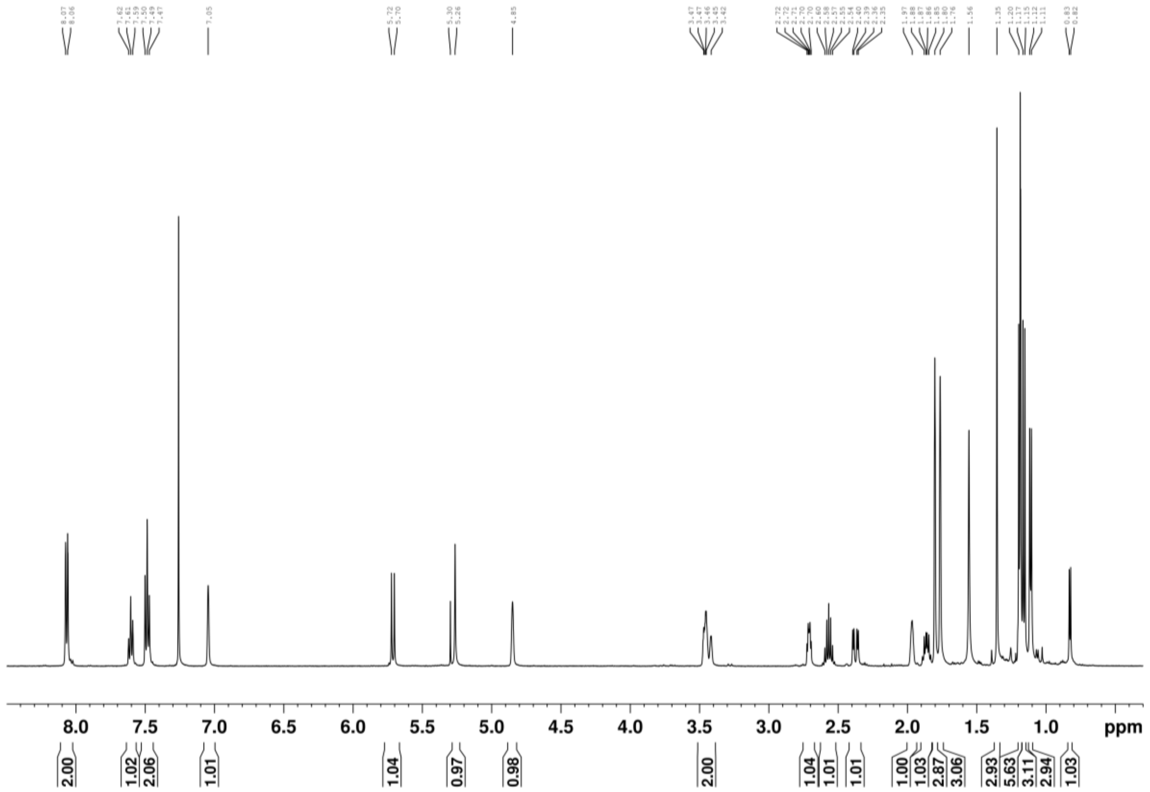

Figure S30: ^{13}C-NMR (125 MHz, $CDCl_3$) spectrum of compound **5**

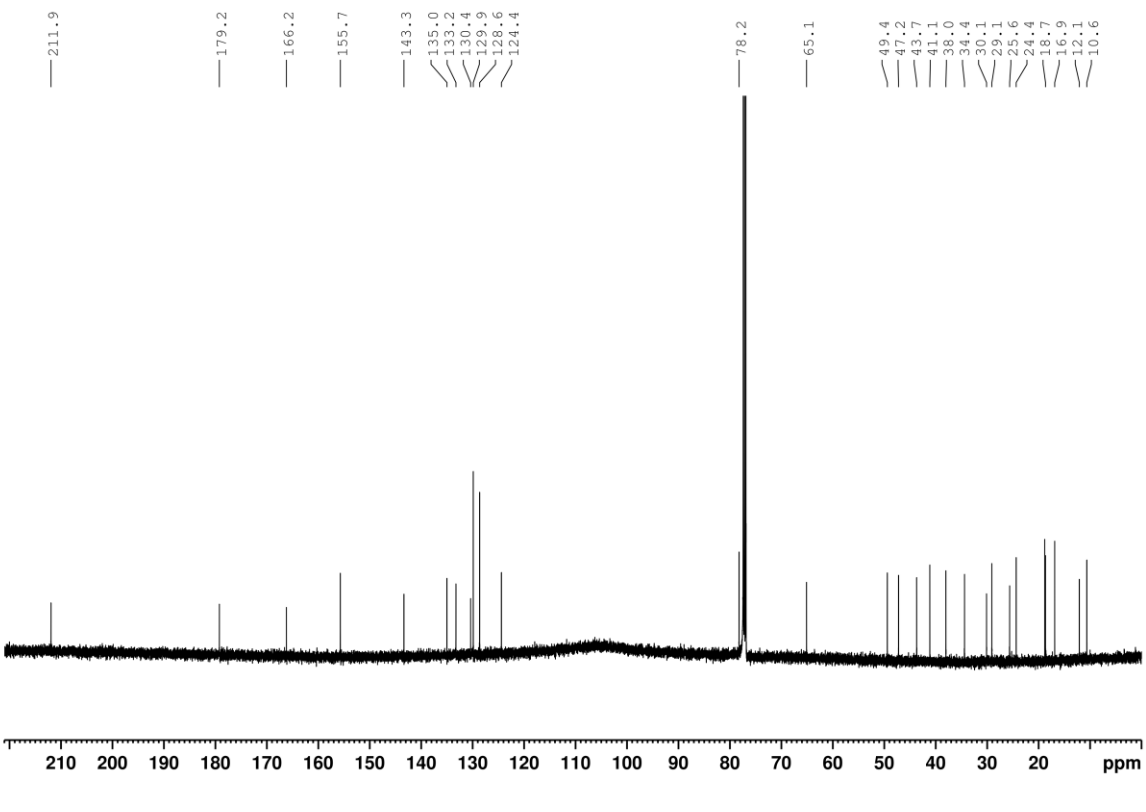

Figure S31: HSQC spectrum of compound **5**

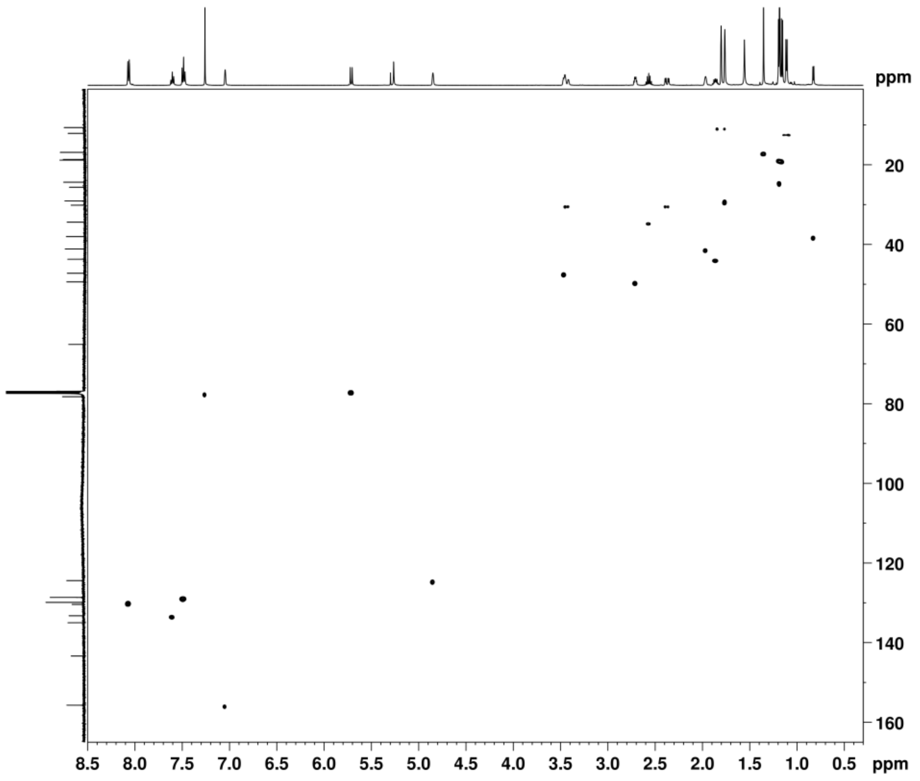

Figure S32: COSY spectrum of compound **5**

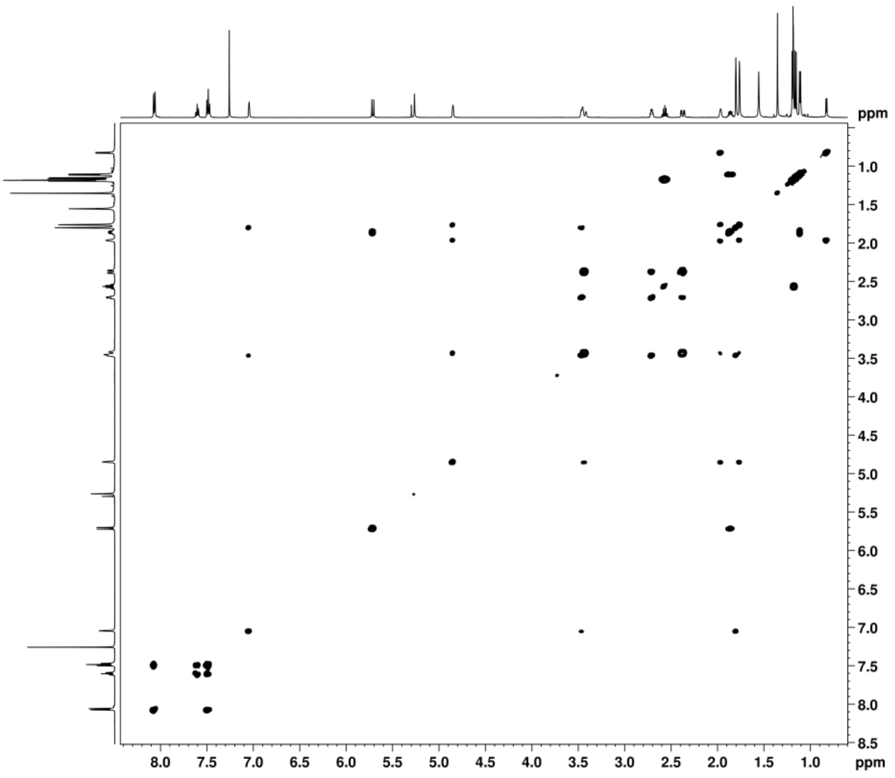

Figure S33: HMBC spectrum of compound **5**

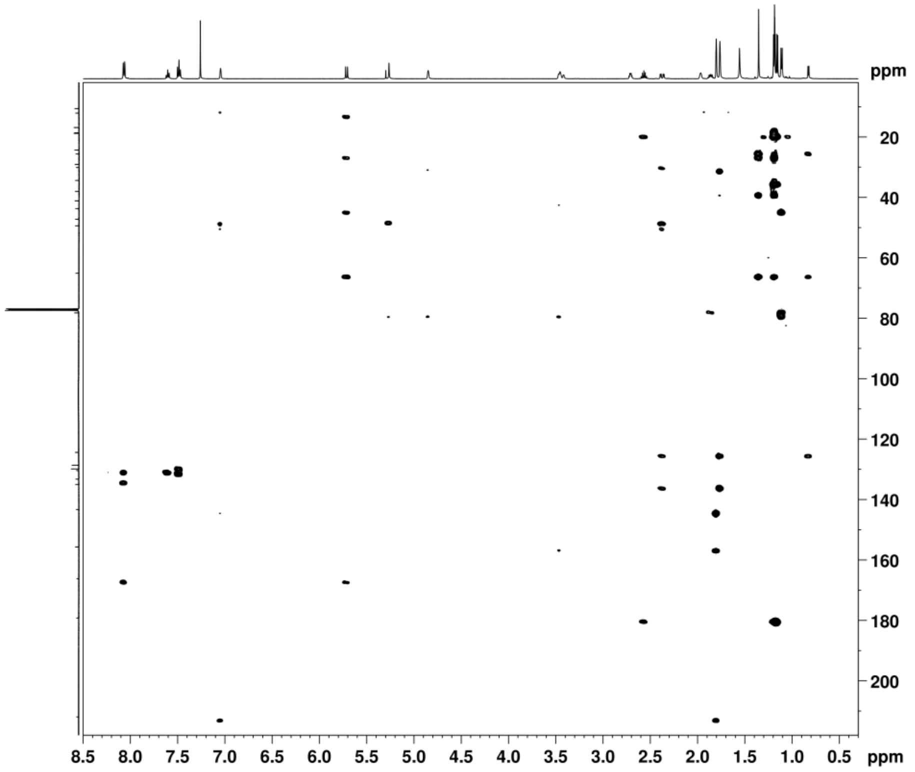

Figure S34: Mass spectrum of compound **5**

Qualitative Compound Report

**Data File**	GK_EGK-3-10-2-1_MK_70V_pos1.d	**Sample Name**	Unavailable
**Sample Type**	Unavailable	**Position**	Unavailable
**Instrument Name**	Unavailable	**User Name**	Unavailable
**Acq Method**		**Acquired Time**	Unavailable
**IRM Calibration Status**	Success	**DA Method**	Default.m
**Comment**	Sample information is unavailable		

Compound Table

Compound Label	RT	Mass	Abund	Formula	Tgt Mass	Diff (ppm)
Cpd 1: C31 H38 O6	0.382	506.2651	5856	C31 H38 O6	506.2668	-3.48

Compound Label	*m/z*	RT	Algorithm	Mass
Cpd 1: C31 H38 O6	507.2737	0.382	Find By Formula	506.2651

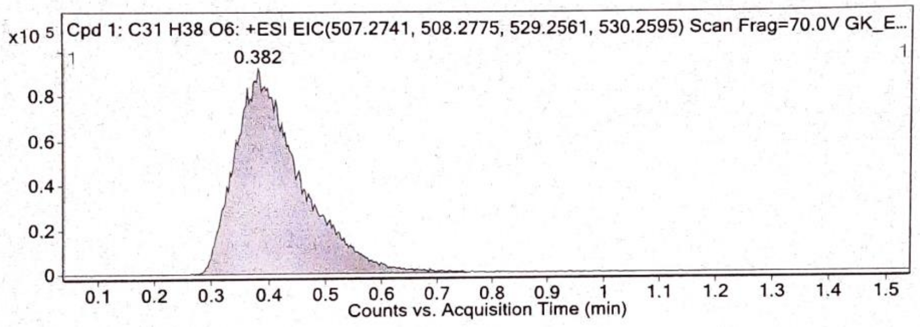

Figure S35: ^{1}H-NMR (500 MHz, $CDCl_3$) spectrum of compound **6**

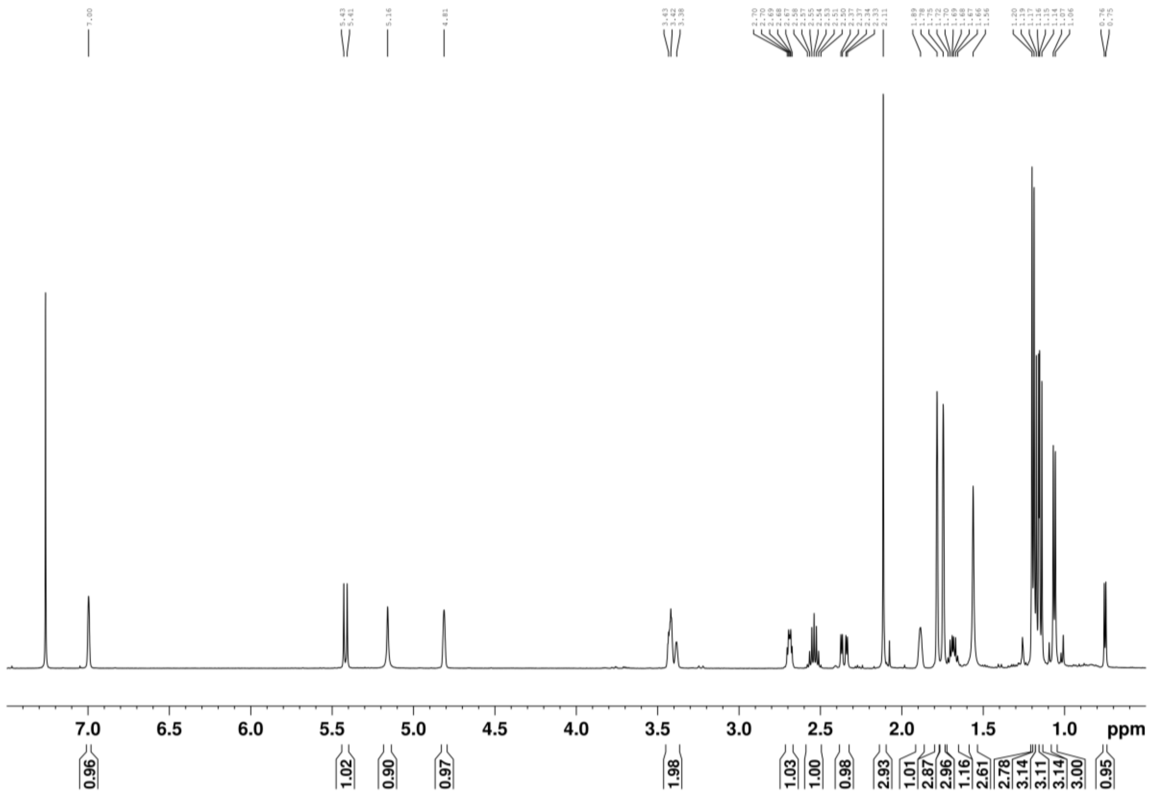

Figure S36: ^{13}C-NMR (125 MHz, $CDCl_3$) spectrum of compound **6**

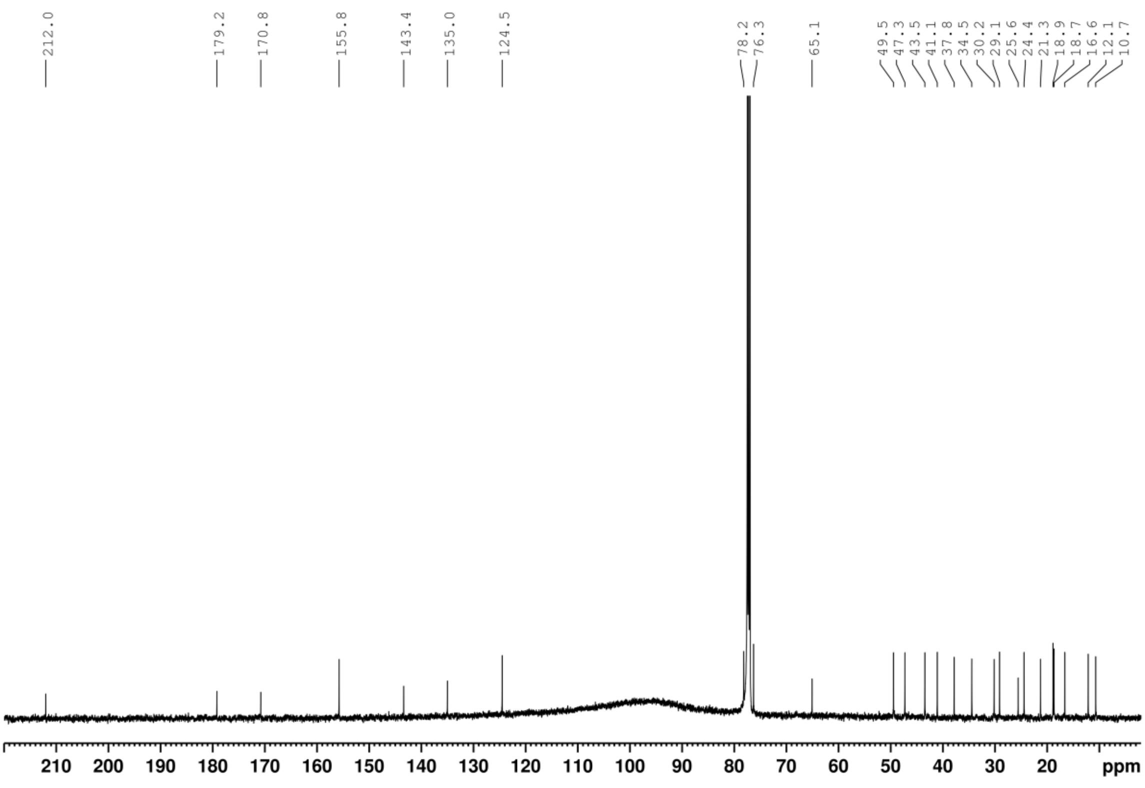

Figure S37: HSQC spectrum of compound **6**

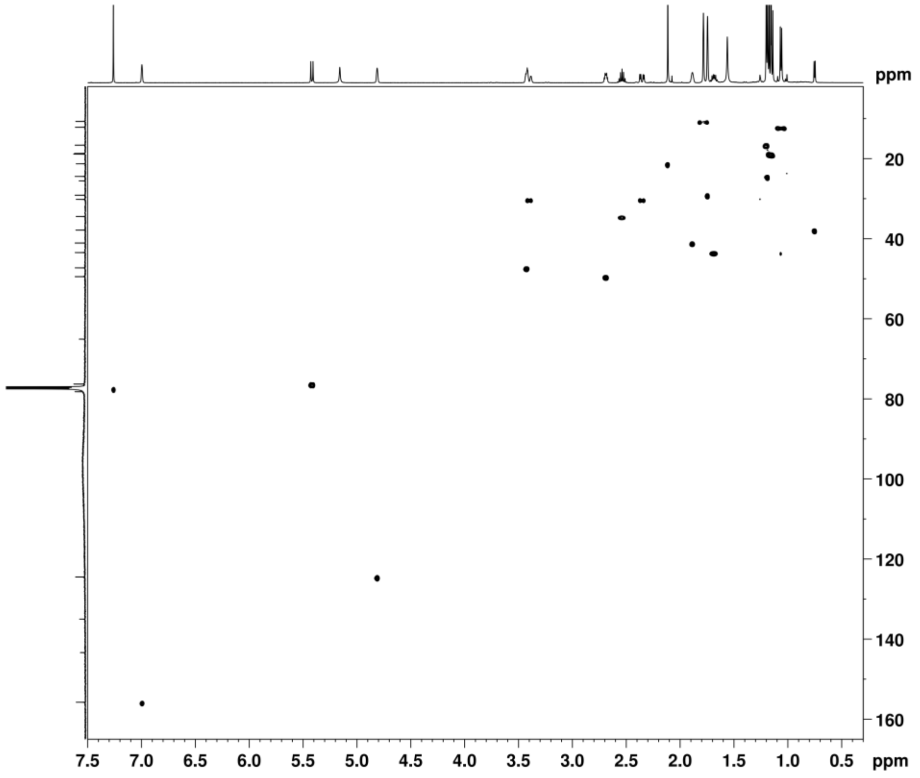

Figure S38: COSY spectrum of compound **6**

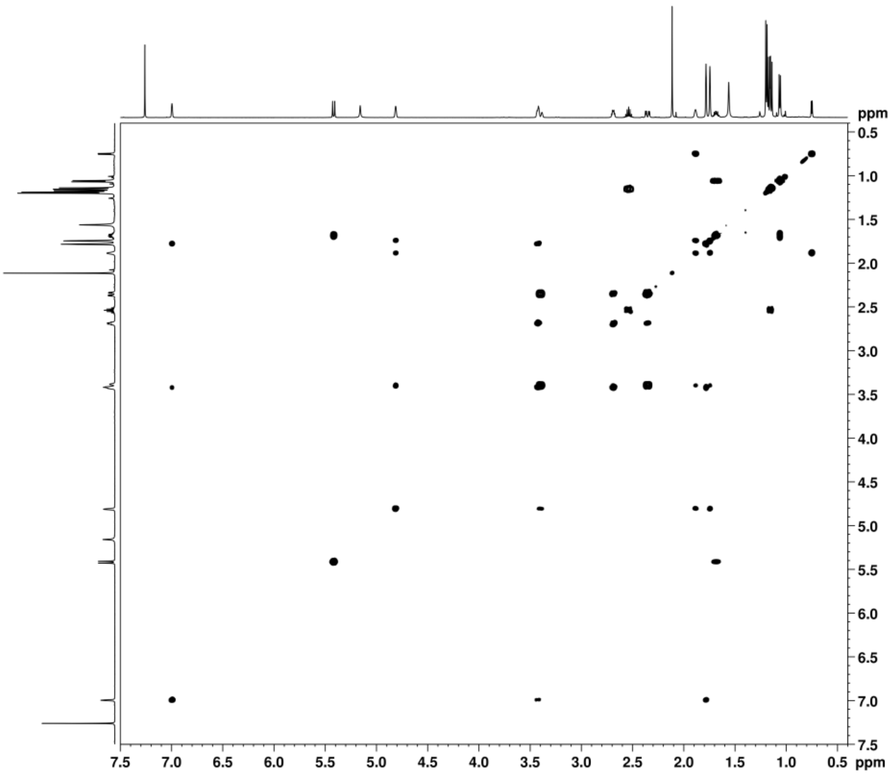

Figure S39: HMBC spectrum of compound **6**

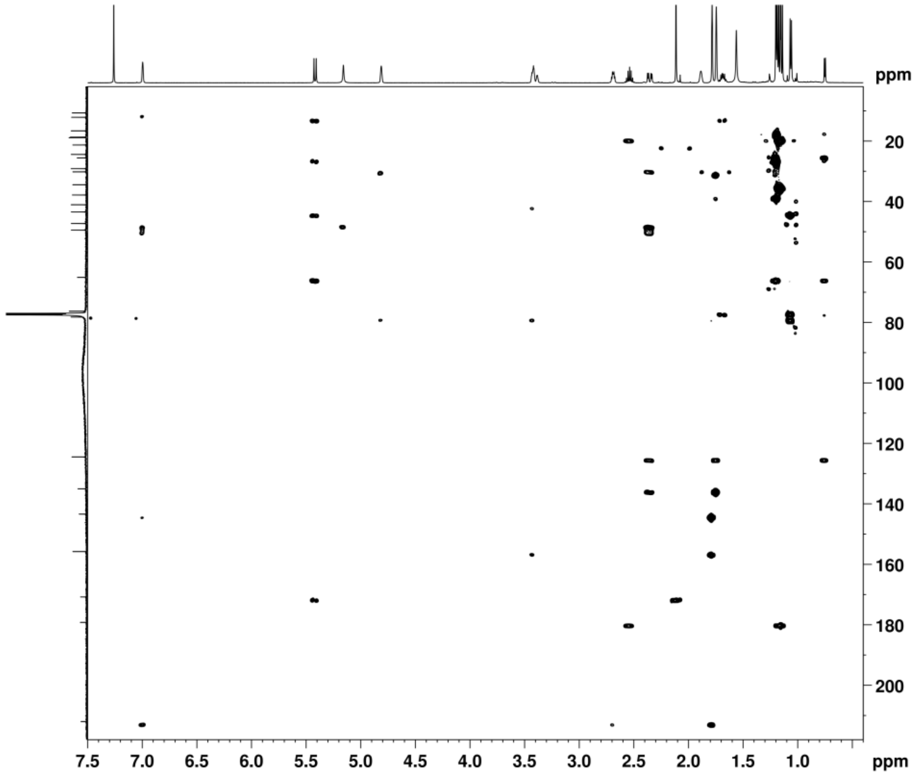

Figure S40: NOESY spectrum of compound **6**

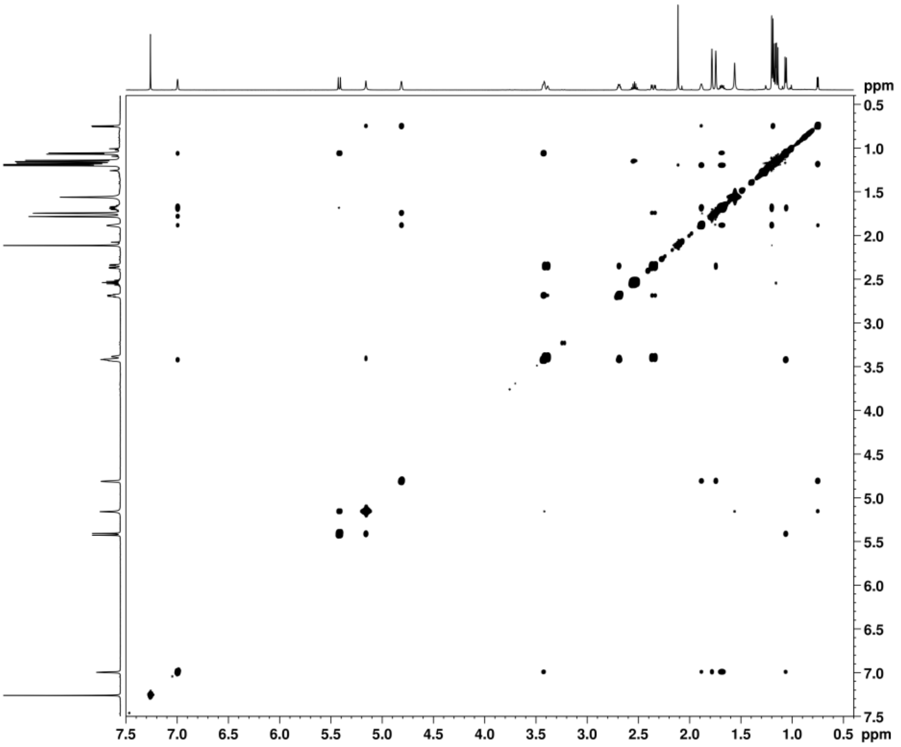

Figure S41: Mass spectrum of compound **6**

Qualitative Compound Report

**Data File**	GK_EGK-3-10-4-1_MK_70V_pos1.d	**Sample Name**	EGK-3-10-4-1
**Sample Type**	Sample	**Position**	P1-B5
**Instrument Name**	DE1517B001	**User Name**	
**Acq Method**	Odredjivanje MM_AF_70V_pos.m	**Acquired Time**	7/3/2019 9:53:28 AM
**IRM Calibration Status**	Success	**DA Method**	Default.m
**Comment**			

**Sample Group**		**Info.**	
**Stream Name**	LC 1	**Acquisition SW Version**	6200 series TOF/6500 series Q-TOF B.06.01 (B6157)

Compound Table

Compound Label	RT	Mass	Abund	Formula	Tgt Mass	Diff (ppm)
Cpd 1: C26 H36 O6	0.381	444.2506	9797	C26 H36 O6	444.2512	-1.24

Compound Label	*m/z*	RT	Algorithm	Mass
Cpd 1: C26 H36 O6	467.2401	0.381	Find By Formula	444.2506

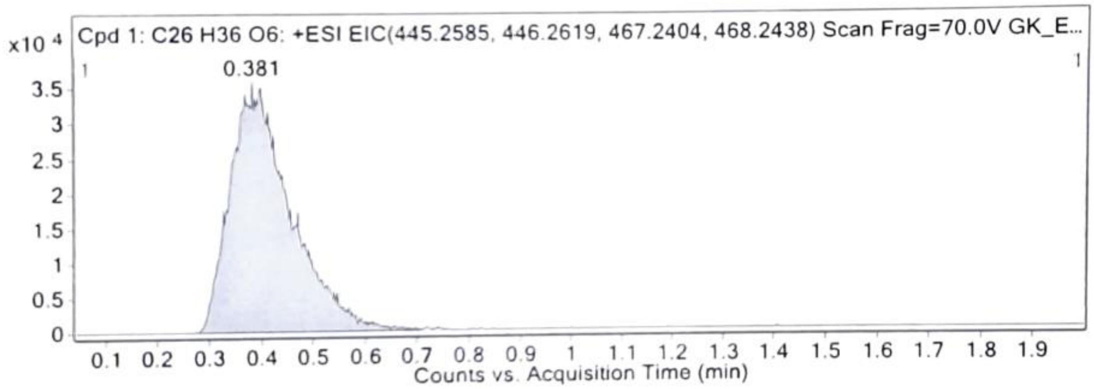

Figure S42: ^{1}H-NMR (500 MHz, $CDCl_3$) spectrum of compound **7**

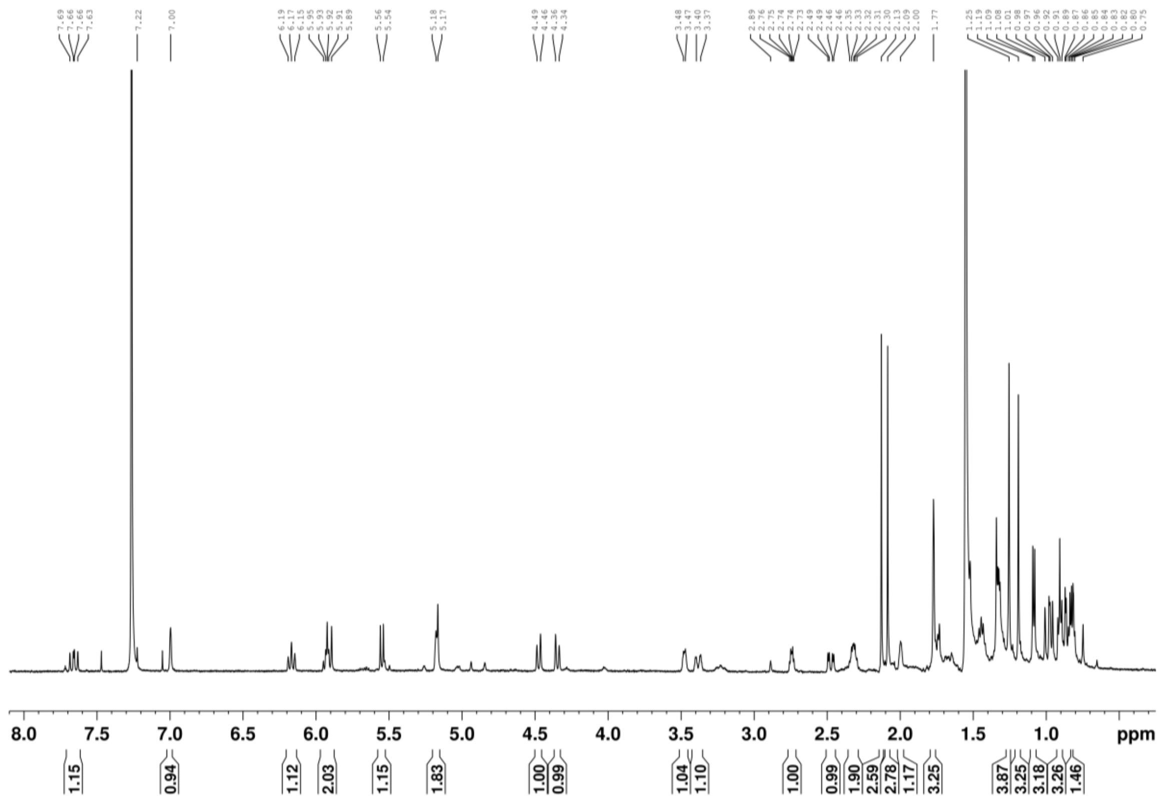

Figure S43: ^{13}C-NMR (125 MHz, $CDCl_3$) spectrum of compound **7**

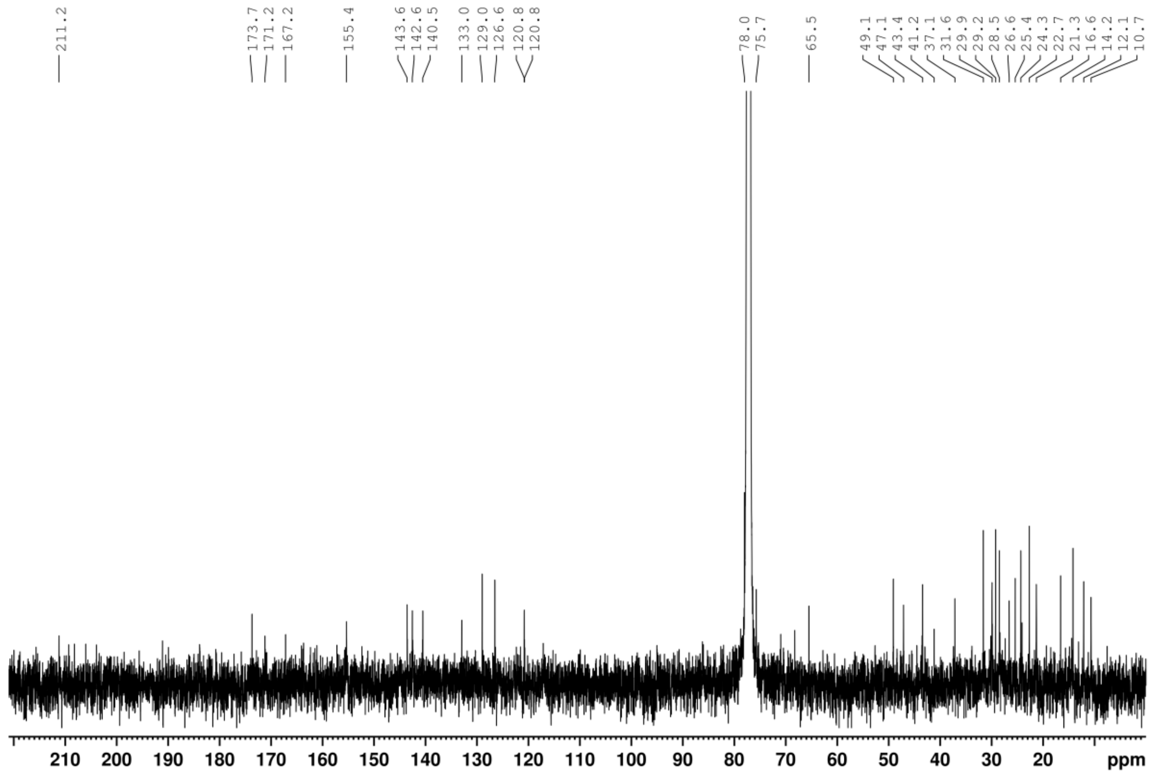

Figure S44: HSQC spectrum of compound **7**

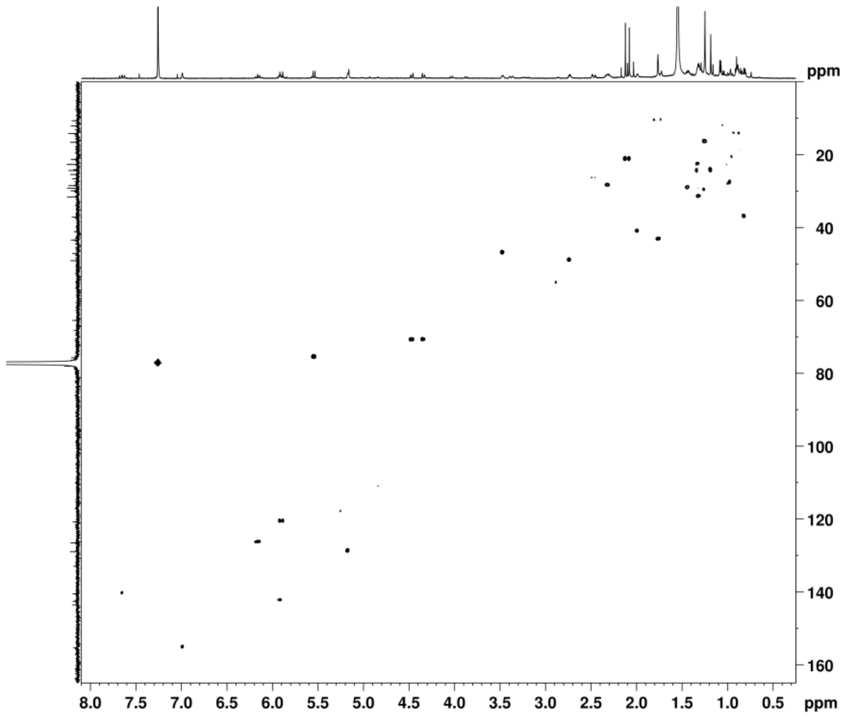

Figure S45: COSY spectrum of compound **7**

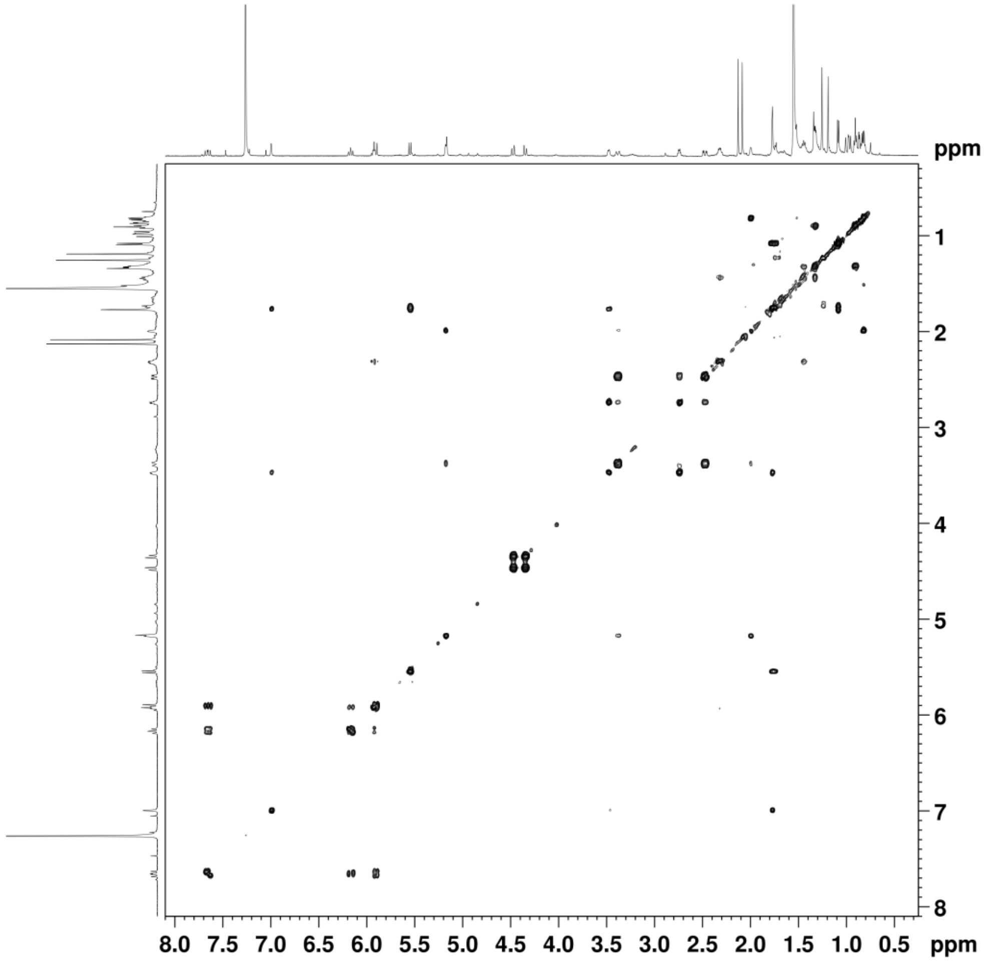

Figure S46: HMBC spectrum of compound **7**

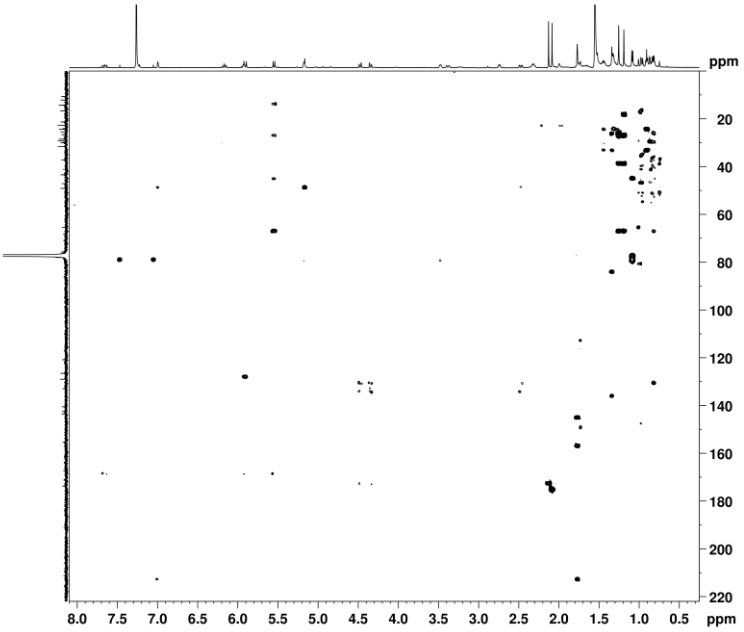

Figure S47: NOESY spectrum of compound **7**

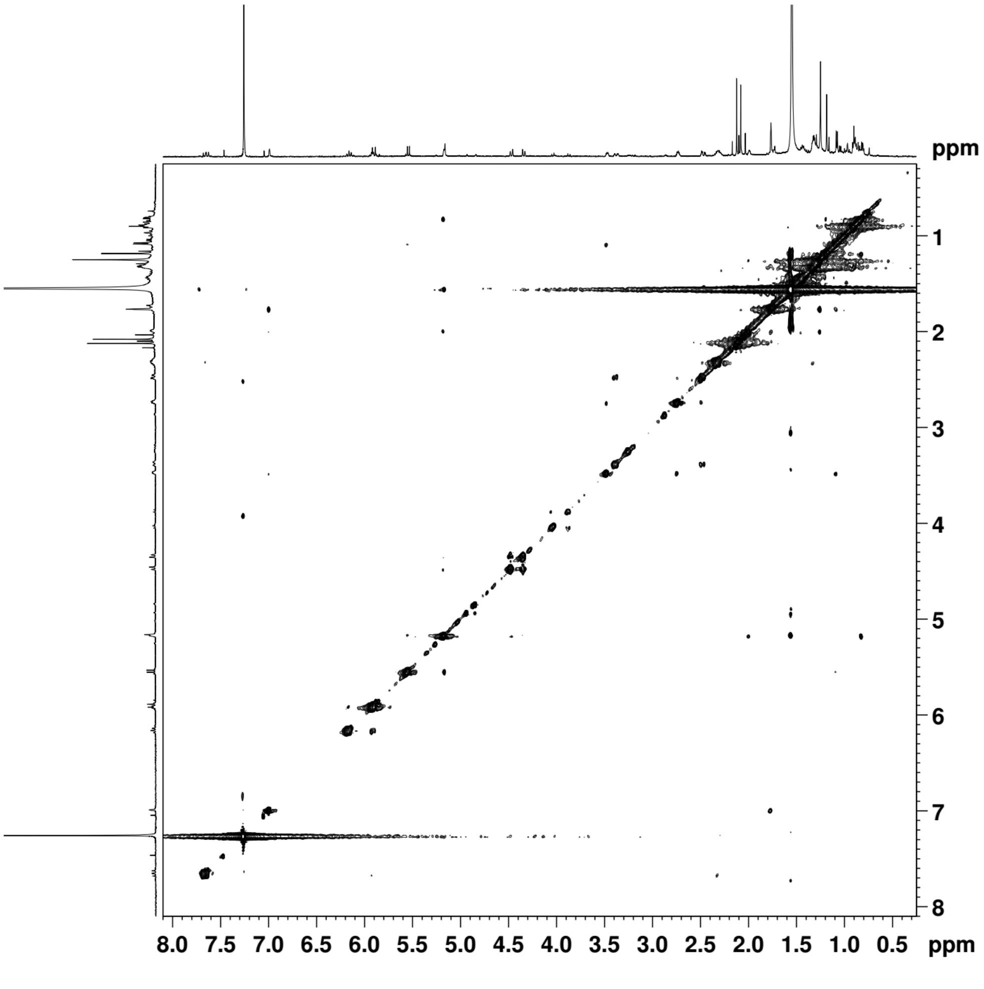

Figure S48: Mass spectrum of compound **7**

Qualitative Compound Report

**Data File**	GK_EGK-4-11-3-3_MK_70V_pos2.d	**Sample Name**	EGK-4-11-3-3
**Sample Type**	Sample	**Position**	P1-B9
**Instrument Name**	DE1517B001	**User Name**	
**Acq Method**	Odredjivanje MM_AF_70V_pos.m	**Acquired Time**	7/3/2019 10:40:47 AM
**IRM Calibration Status**	Success	**DA Method**	Default.m
**Comment**			

**Sample Group**		**Info.**	
**Stream Name**	LC 1	**Acquisition SW Version**	6200 series TOF/6500 series Q-TOF B.06.01 (B6157)

Compound Table

Compound Label	RT	Mass	Abund	Formula	Tgt Mass	Diff (ppm)
Cpd 1: C34 H46 O8	0.383	582.3193	12257	C34 H46 O8	582.3193	0.06

Compound Label	*m/z*	RT	Algorithm	Mass
Cpd 1: C34 H46 O8	583.3265	0.383	Find By Formula	582.3193

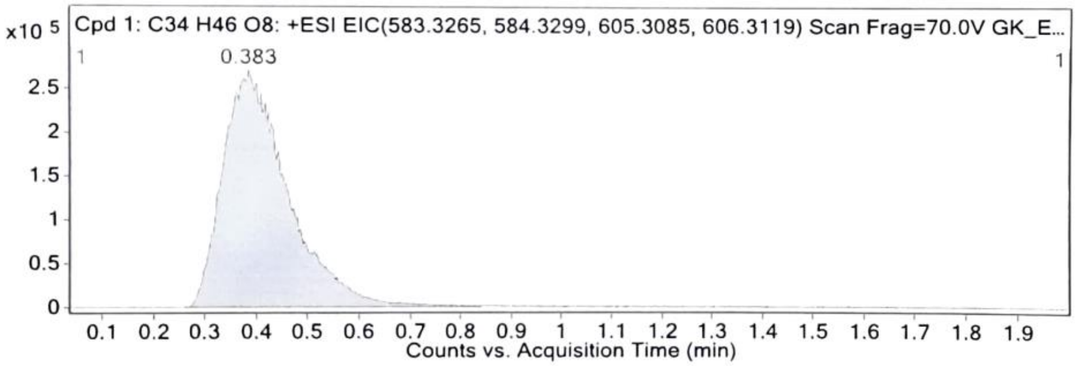

Figure S49: ^{1}H-NMR (500 MHz, $CDCl_3$) spectrum of compound **8**

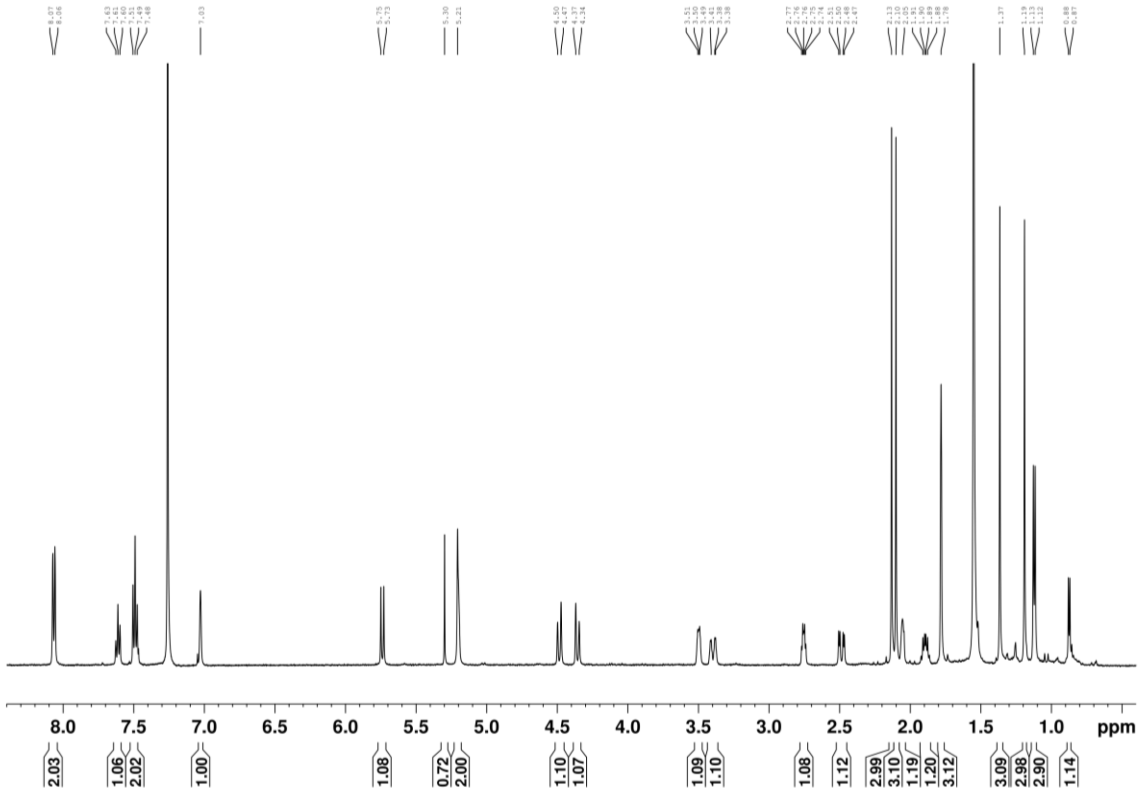

Figure S50: ^{13}C-NMR (125 MHz, $CDCl_3$) spectrum of compound **8**

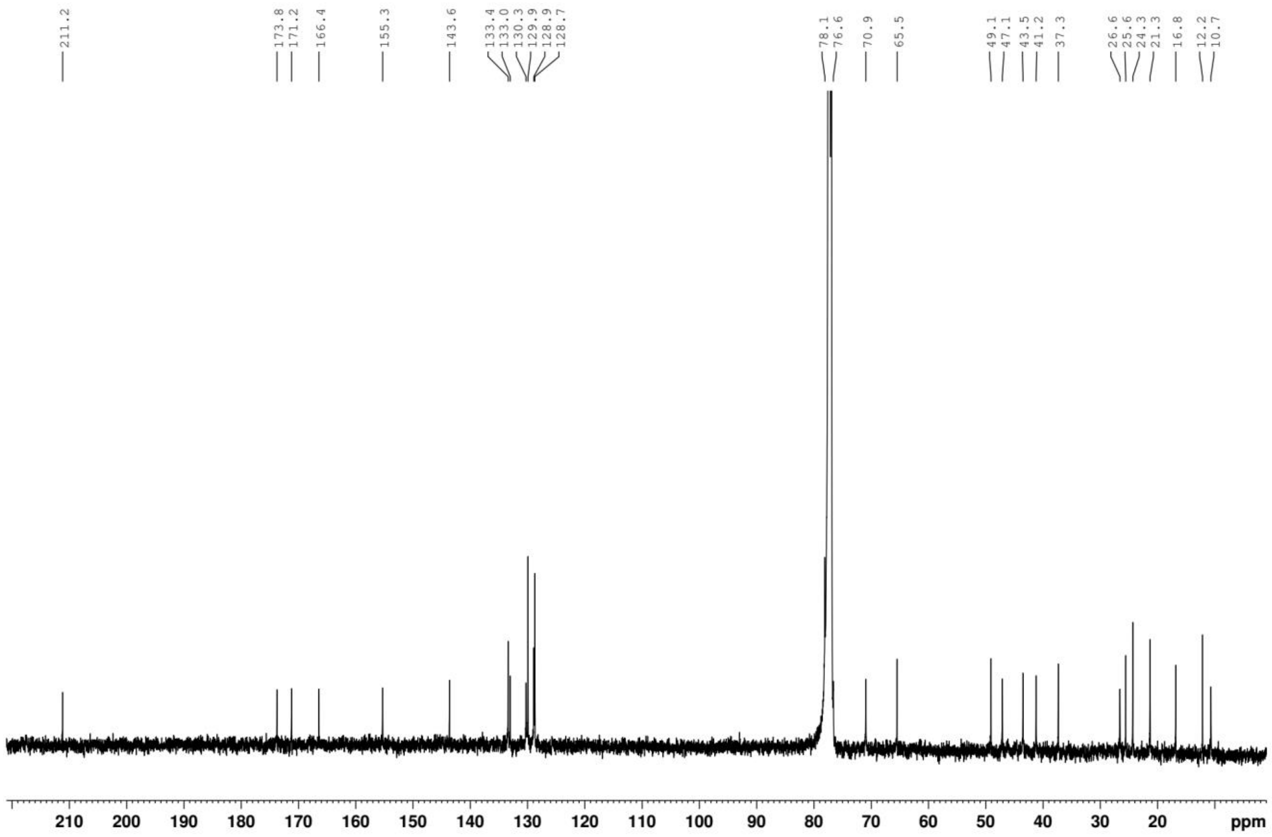

Figure S51: HSQC spectrum of compound **8**

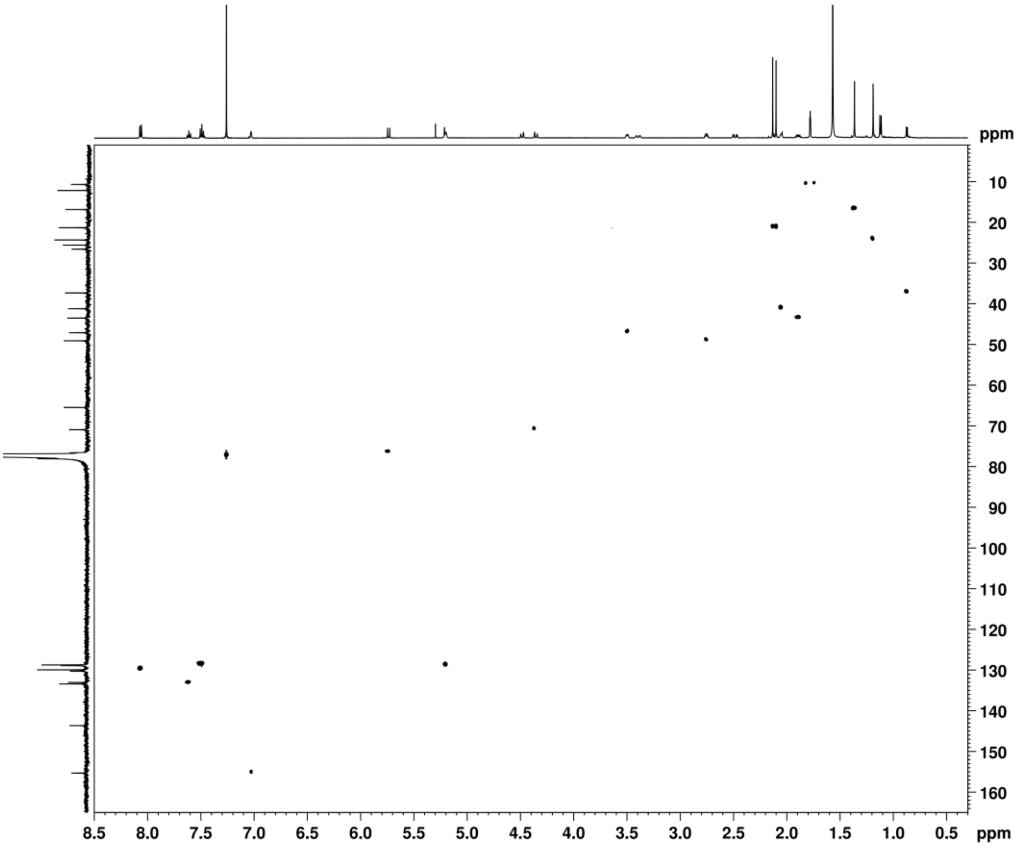

Figure S52: COSY spectrum of compound **8**

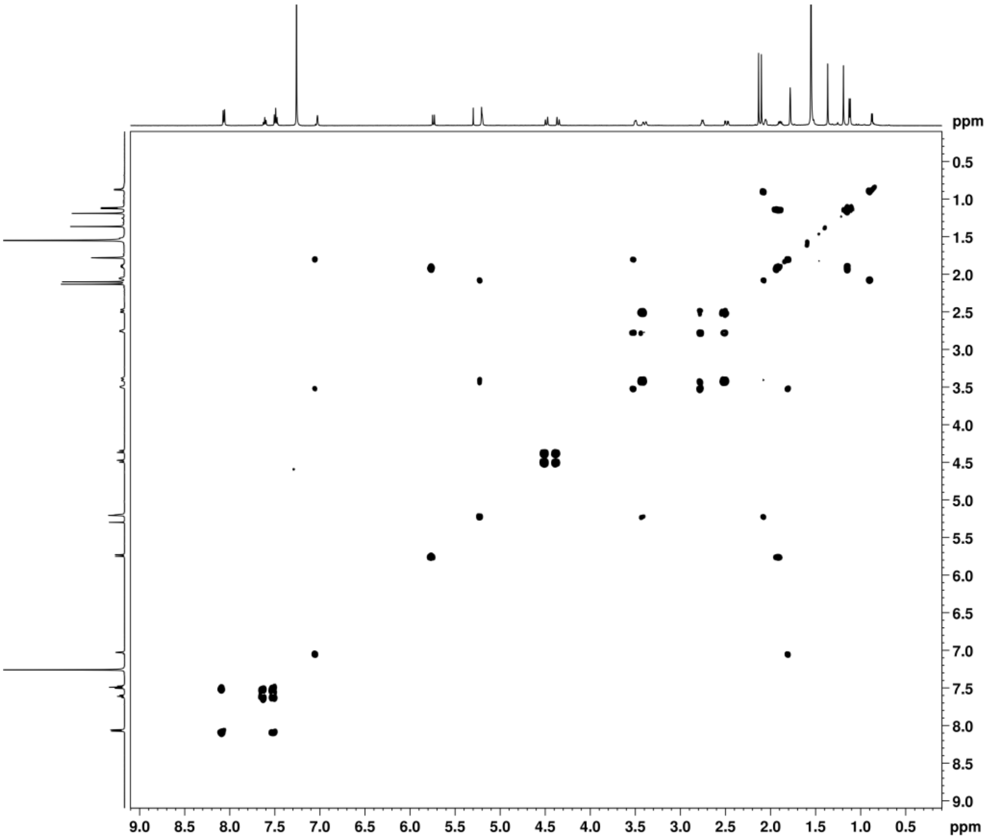

Figure S53: HMBC spectrum of compound **8**

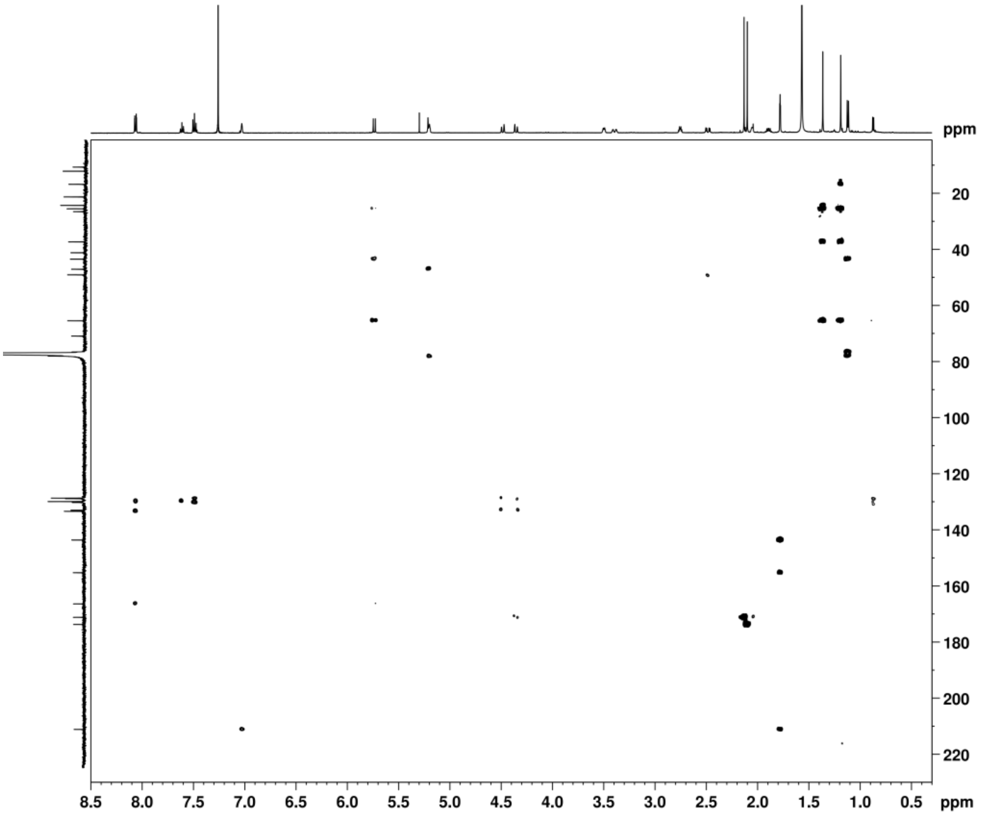

Figure S54: NOESY spectrum of compound **8**

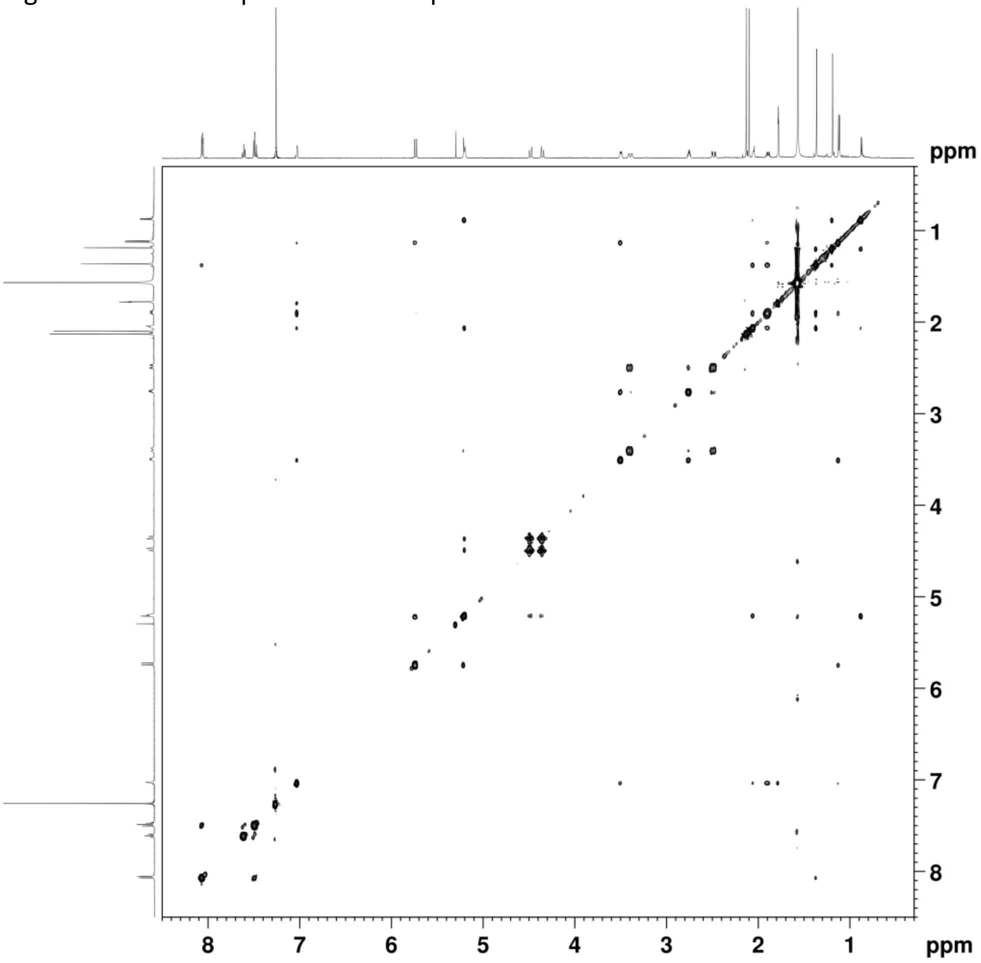

Figure S55: Mass spectrum of compound **8**

Qualitative Compound Report

**Data File**	GK_EGK-4-11-10-1_MK_70V_pos1.d	**Sample Name**	EGK-4-11-10-1
**Sample Type**	Sample	**Position**	P1-C3
**Instrument Name**	DE1517B001	**User Name**	
**Acq Method**	Odredjivanje MM_AF_70V_pos.m	**Acquired Time**	7/3/2019 10:14:58 AM
**IRM Calibration Status**	Success	**DA Method**	Default.m
**Comment**			

**Sample Group**		**Info.**	
**Stream Name**	LC 1	**Acquisition SW Version**	6200 series TOF/6500 series Q-TOF B.06.01 (B6157)

Compound Table

Compound Label	RT	Mass	Abund	Formula	Tgt Mass	Diff (ppm)
Cpd 1: C31 H36 O8	0.388	536.2407	19397	C31 H36 O8	536.241	-0.66

Compound Label	*m/z*	RT	Algorithm	Mass
Cpd 1: C31 H36 O8	537.248	0.388	Find By Formula	536.2407

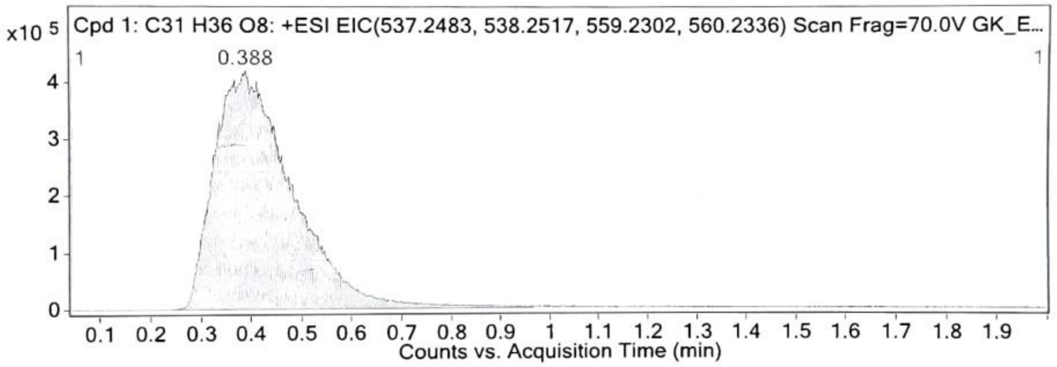

Supplement: Supplementary file 1 [file molecules-30-01452-s001.zip › molecules-3525064-supplementary.pdf]
